# AFP assistant: a retrieval-augmented generation and large language model-powered multilingual polio chatbot for low-resource language communities

**DOI:** 10.1038/s41746-026-02944-4

**Published:** 2026-06-30

**Authors:** Gelane Biru, Honey Gemechu, Firanol Teshome, Eyerusalem Gebremeskel, Hundessa Daba Nemomssa, Kokeb Dese, Melaku Tilahun, Karrasaaqii Bulti Wakgari, Nardos Bisrat, Gelan Ayana, Jude Kong

**Affiliations:** 1 School of Biomedical Engineering, Jimma Institute of Technology, Jimma University, Jimma, Ethiopia; 2 Global South Artificial Intelligence for Pandemic and Epidemic Preparedness and Response Network (AI4PEP), Toronto, ON Canada; 3 Faculty of Computing and Informatics, Jimma Institute of Technology, Jimma University, Jimma, Ethiopia; 4 Department of Educational Technology and Information Management, Mattu University, Mattu, Ethiopia; 5 Department of Biomedical Engineering, Gannon University, Erie, PA USA; 6 Artificial Intelligence & Mathematical Modeling Lab (AIMMLab), Dalla Lana School of Public Health, University of Toronto, Toronto, ON Canada; 7 Department of Mathematics, Bahen Centre for Information Technology, University of Toronto, Toronto, ON Canada; 8 Africa-Canada Artificial Intelligence and Data Innovation Consortium (ACADIC), Toronto, ON Canada

**Keywords:** Health care, Mathematics and computing

## Abstract

Acute flaccid paralysis (AFP) surveillance in Ethiopia remains limited due to hard-to-reach populations, restricted healthcare access, low public awareness, and persistent underreporting within community-based surveillance (CBS) systems. Contributing to this are inadequate training of community reporters, communication barriers, and cultural beliefs and misinformation that further hinder timely case detection. Here, AFP Assistant, a multilingual large language model powered chatbot, is developed to strengthen CBS through improved health communication and real-time reporting. The system supports Amharic, Afaan Oromo, and English to enhance accessibility and inclusivity. A curated dataset derived from literature, guidelines, and community interviews was translated and validated by experts to ensure clinical and cultural accuracy. The system integrates supervised fine-tuning with retrieval-augmented generation (RAG) to deliver accurate, context-aware responses. Trained on 468 question–answer pairs using pretrained Gemini 2.5-flash, the model achieved an accuracy of 0.90 and a loss of 0.31. The RAG framework improved retrieval relevance, grounding, and response efficiency. Evaluation through in-house testing and human annotation using a structured rubric demonstrated robust performance across tasks and languages, while user feedback confirmed usability and relevance. These findings highlight the potential of AI-driven chatbots to enhance AFP surveillance and equitable health communication in low-resource settings.

## Introduction

Polio is a highly contagious infectious disease caused by the poliovirus, which can lead to sudden muscle weakness and, in severe cases, permanent paralysis^1–4^. This paralysis is referred to as acute flaccid paralysis (AFP), defined as the abrupt onset of weakness or paralysis with reduced muscle tone, most often occurring in children, or in any paralytic illness where poliomyelitis is suspected^5,6^. Although poliovirus is the most notable cause, AFP can arise from a wide range of etiologies, including other infectious agents (e.g., enteroviruses), autoimmune disorders (e.g., Guillain–Barré syndrome, Transverse Myelitis), and neuromuscular conditions such as myasthenia gravis or botulism^1,7–10^. Its clinical manifestations are diverse, often presenting as facial droop, limb weakness, swallowing or speech difficulties, and respiratory compromise, all of which require urgent medical attention. To eradicate the disease, the Global Polio Eradication Initiative (GPEI) was established and outlined four endgame strategies aimed at supporting eradication efforts while enhancing the detection of silent circulation of vaccine-derived polioviruses (VDPVs)^11,12^. Among these, high-quality AFP surveillance is recognized as the gold standard for detecting and reporting polio cases^13,14^. One important approach within AFP surveillance is community-based surveillance (CBS), which leverages community volunteers (CVs) and local leaders to actively identify and report suspected AFP cases through systematic case finding^15^. CBS for AFP is particularly crucial in hard-to-reach pastoral and semi-pastoral areas, where local engagement plays a vital role in timely case identification and reporting^16,17^. Despite its advantage, community-based AFP surveillance in many regions faces persistent challenges, including delayed detection and reporting, limited community awareness, inadequate health infrastructure, low immunity in underserved populations, high population mobility, security constraints, and surveillance performance gaps, especially in underserved communities^18–21^.

In Ethiopia, CBS for AFP serves as a vital mechanism for early detection, reporting, and investigation of poliomyelitis cases^22^. However, its overall effectiveness remains limited by systemic and contextual barriers, including inadequate public health infrastructure, political instability, weak communication networks, volunteer demotivation, low health literacy, and inadequate access to reliable information^23,24^. Deep-rooted cultural misconceptions and misinformation further complicate the situation^25,26^. In many communities, AFP is attributed to supernatural causes such as witchcraft or spiritual afflictions. As a result, parents of paralyzed children often seek help from traditional bone setters or religious institutions rather than formal healthcare facilities. A key barrier to effective detection is the absence of a community-level knowledge base about the disease. This knowledge gap reduces the sensitivity of AFP surveillance, as many community members cannot differentiate between polio and other causes of paralysis.

To address persistent challenges in CBS for AFP, a range of multifaceted interventions have been introduced. These include intensive training and capacity-building programs, alongside the expansion of surveillance activities into hard-to-reach and conflict-affected areas, while maintaining both sensitivity and specificity^10^. Central to these efforts has been the structured engagement of community members, including volunteers, local leaders, and frontline health workers, which has significantly improved case detection and reporting^18^. Their participation has fostered trust, heightened community awareness, enhanced the timeliness of notifications, and strengthened the overall effectiveness and sustainability of the surveillance system. In recent years, these community-driven approaches have been complemented by technological innovations designed to bridge systemic gaps within the AFP surveillance framework^27–29^. Notable examples include the deployment of mobile applications to facilitate timely and accurate data collection^30,31^, and the use of educational campaigns disseminated via social media and digital platforms to reinforce awareness and sustain community engagement^32^. Despite these advancements, critical gaps remain, highlighting the need for more comprehensive strategies. In particular, the integration of emerging digital technologies such as artificial intelligence (AI) holds considerable potential for enhancing predictive analytics, automating case detection, and enabling intelligent information dissemination. Leveraging such innovations could further strengthen the sensitivity, timeliness, and responsiveness of AFP surveillance, ensuring greater resilience and effectiveness in diverse settings.

Recent advances in AI, especially in large language models (LLMs), have accelerated the growth of conversational AI, with chatbots emerging as powerful conversational entities in healthcare^33^. Several studies have explored their development across diverse health applications, including medication support, disease monitoring, mental health counseling, symptom assessment, and patient assistance in accessing trustworthy health information. For instance, one study introduced a specialized chatbot for ophthalmologic consultations, particularly macular degeneration, using a bidirectional encoder representation from transformers (BERT), gated recurrent unit (GRU), and term frequency–inverse document frequency (TF-IDF) based retriever–reader model^34^, while another work developed an automated medical chatbot to streamline healthcare communication through AI-driven conversational interfaces built on natural language processing (NLP) techniques^35^. Medibot similarly aims to enhance healthcare accessibility and user interaction by integrating NLP with mapping technology^36^, and the proposed E-Health Bot employs advanced NLP algorithms to enable more human-like interactions^37^. In the mental health domain, researchers have integrated models such as efficiently learning an encoder that classifies token replacements accurately (ELECTRA) and BERT into chatbots to improve counseling support^38^, whereas other work leverages LLMs, particularly generative pre-trained transformer (GPT)-4, to more faithfully replicate patient-provider conversations, thereby enhancing diagnosis and treatment recommendations^39^. Another research in the same domain explores a consultation chatbot to bridge the lack of awareness and limited knowledge about depression using NLP^40^. Additionally, cutting-edge NLP techniques using large language model meta-AI (LLaMA) have been applied to build medical chatbots capable of handling a broad range of medical inquiries^41^. In the African context, emerging research has begun to address the unique challenges of low-resource languages and settings. For example, studies in Afaan Oromo have explored symptom-based specialist recommendation systems, contributing to the development of domain-specific corpora and supervised learning approaches tailored to local linguistic contexts^42^. Similarly, a Swahili-language medical chatbot (Lueji) adopts a hybrid fine-tuning and retrieval-augmented generation (RAG) approach to address low-resource constraints^43^.

For infectious diseases, particularly in the context of COVID-19, chatbots have been extensively used to disseminate health information on symptoms, precautionary measures, and available medical treatments^44–46^. Beyond information delivery, these systems have also played a critical role in combating misinformation and limiting the spread of false or misleading health content^47^. Extending this application to broader public health contexts, tools such as AIDA^48^, a general-purpose chatbot developed in collaboration with UNICEF has been designed to provide reliable information on childhood vaccination. However, almost all health-sector chatbots are developed for resource-rich languages, particularly English, which limits access for communities in low-resource language settings where language barriers persist. While recent systems often fine-tune LLMs for domain-specific tasks, leveraging their ability to predict and assemble statistically likely word sequences to answer complex questions and follow intricate instructions^49^, customization for low-resource languages remains challenging due to scarce datasets, high computational costs, and persistent research gaps^50,51^. The underrepresentation of low-resource languages, which are less studied and less digitized, further hinders advancements in NLP compared to English^52–55^.

Moreover, though many healthcare chatbots have been developed in recent years, a critical gap persists in addressing infectious diseases such as polio, particularly in low-resource settings where community awareness and early detection are essential. To bridge this gap, this study presents the development of AFP Assistant, a multilingual, AI-powered chatbot built on a hybrid architecture that integrates LLM fine-tuning with RAG to deliver accurate, context-aware, and continuously updated responses. Unlike existing tools, the key contribution of this work is the development of a multilingual assistant supporting Amharic and Afaan Oromo alongside English, enabling access for users in low-resource language settings and promoting health information equity. In addition to general awareness, the system integrates AFP surveillance guidelines, locally relevant myths and misconceptions, and contextual public health information related to polio in Ethiopia. The chatbot provides trusted information on symptoms, vaccination, and prevention, guides users in reporting suspected AFP cases, and supports frontline health workers with on-demand, evidence-based resources. By recording queries anonymously and generating aggregated insights, the system contributes to data-driven public health strategies and awareness initiatives. Beyond its technical innovation, the AFP Assistant represents a purposeful advancement toward equitable healthcare access by delivering vital medical information in underserved languages and strengthening CBS systems. More than a digital information tool, it fosters meaningful community engagement, facilitates early case detection, and enables timely reporting, thereby reinforcing Ethiopia’s public health infrastructure and supporting global polio eradication efforts. Its distinctive value lies in bridging language barriers and addressing digital health inequities through an inclusive platform that empowers both health workers and community members. For example, a mother in a remote village who suspects her child may show signs of AFP can interact with the chatbot in her native language, such as Amharic or Afaan Oromo, to receive accurate, culturally and linguistically accessible guidance on symptoms, next steps, and care-seeking pathways. By enabling real-time, context-sensitive communication, the AFP Assistant demonstrates strong potential to enhance disease surveillance, promote informed decision-making, and strengthen preparedness for future public health emergencies in Ethiopia and beyond.

## Results

### Results of language model selection

**Fig. 1:**
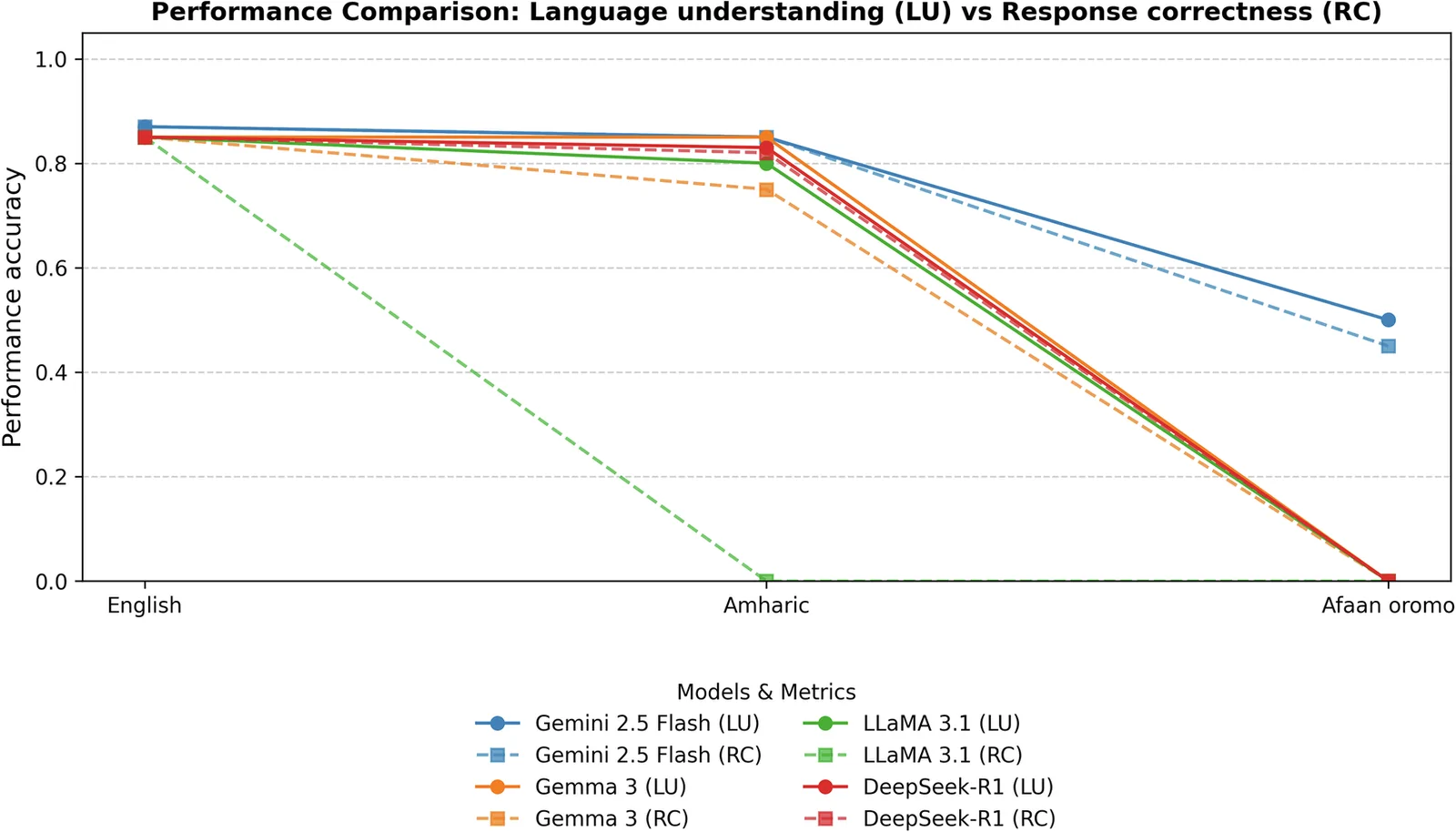
Model performance comparison between LLMs for selection. Performance comparison of LLMs across English, Amharic, and Afaan Oromo in terms of LU and RC. The results show that Gemini 2.5 Flash maintains the highest performance consistency across all three languages, particularly in low-resource contexts. In contrast, LLaMA 3.1 demonstrates a sharp performance drop in non-English languages, while Gemma 3 and DeepSeek-R1 exhibit moderate declines. Generally, Gemini 2.5 Flash demonstrates the highest multilingual robustness and accuracy compared to other models.

Following the screening of candidate models based on research-specific criteria, a comparative evaluation was conducted on Gemma 3, LLaMA 3.1, and DeepSeek-R1 via Hugging Face, as well as Gemini 2.5-flash via Vertex AI. Each model was tested using five benchmark questions per language in English, Amharic, and Afaan Oromo. Language understanding (LU) was defined as the model’s ability to correctly interpret queries in the target language, while response correctness (RC) was assessed by comparing model outputs against reference documents for semantic and factual accuracy. As shown in Fig. 1, all models perform near-optimally in English; however, performance declines in low-resource languages. Gemini 2.5-flash demonstrates the strongest cross-lingual robustness, resulting in its selection for this work. Gemma 3 and DeepSeek-R1 achieve moderate LU but with reduced RC, and LLaMA 3.1 performs poorly in both Amharic and Afaan Oromo.

### Results for the finetuned best model

**Fig. 2:**
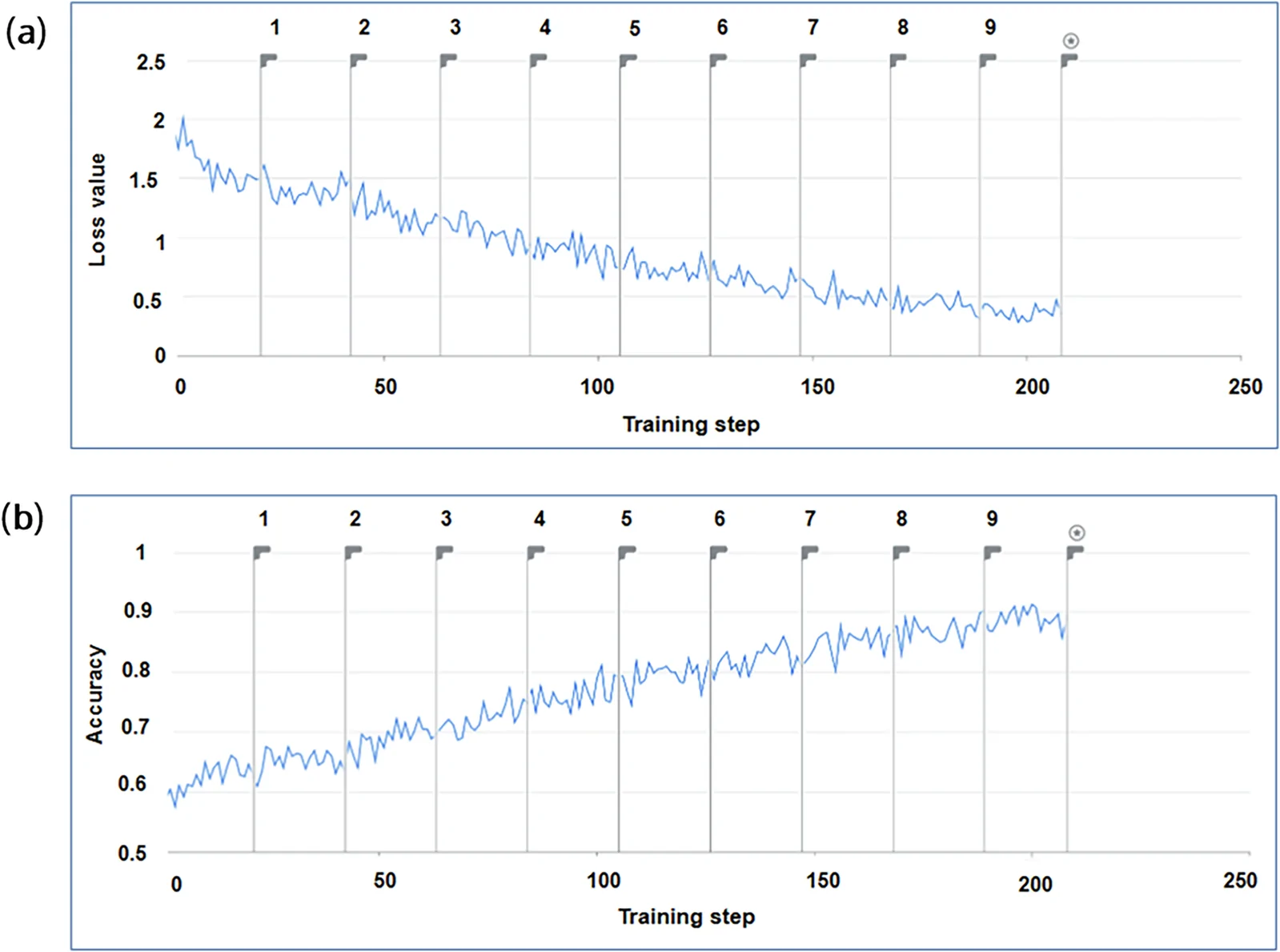
Fine-tuning performance of the Gemini model on the multilingual AFP dataset. **a** Shows the training loss curve across training examples, illustrating a progressive decrease in loss from approximately 2.3 to 0.31, indicating successful model convergence and reduction of prediction errors during fine-tuning. **b** Presents the token-level prediction accuracy reported by Vertex AI during supervised fine-tuning, increasing from approximately 58% to 90% as training progresses. The X-axis in both panels represents the number of training examples processed,while the Y-axis represents loss in **a** and accuracy in **b**.

As shown in Fig. 2, fine-tuning the Gemini model on the polio dataset using Vertex AI led to effective convergence. The loss curve (a) decreased steadily from approximately 2.3–0.31, indicating consistent error reduction throughout the training process despite minor batch-related fluctuations. Simultaneously, the accuracy curve (b) increased from 58% to 90%. In this context, the reported accuracy represents the token-level prediction accuracy measured during SFT, reflecting how often the model correctly predicts the next token in the reference response within the provided training and validation datasets. This metric is automatically reported by Vertex AI during training and serves as an indicator of the model’s learning progress and improved predictive capability. These trends suggest that the fine-tuned Gemini model successfully learned domain-specific patterns relevant to polio and AFP-related information. However, it should be noted that this accuracy reflects training-time performance on the training and validation splits and does not represent evaluation on a separate held-out test dataset.

### Results for retrieval-augmented generation

**Fig. 3:**
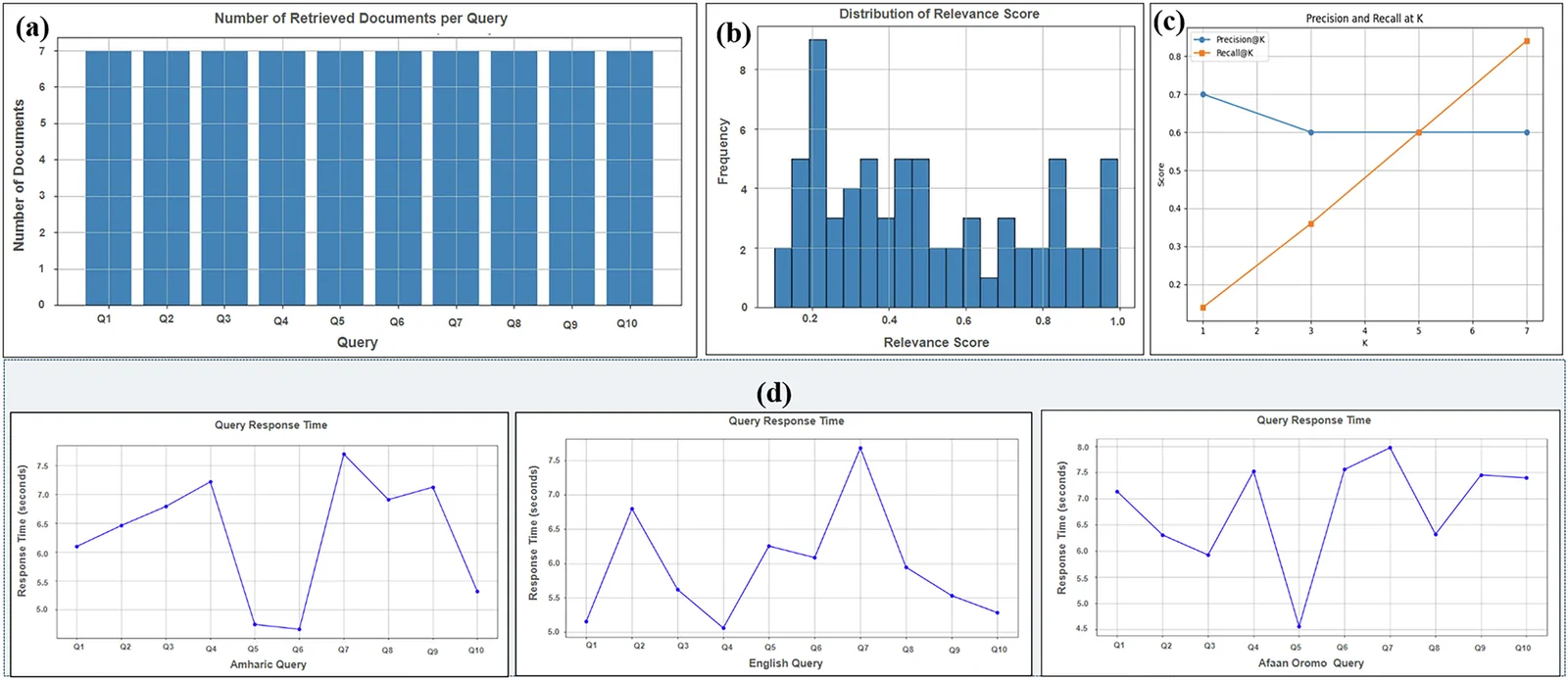
Retrieval performance and query response analysis of the multilingual RAG system. RAG performance evaluation metrics results for **a** indicating the number of retrieved documents per query and **b** distribution of relevance score for queries asked in English, **c** precision and recall across different values of k, and **d** show the response time of the system in Amharic, English, and Afaan Oromo queries, respectively.

The evaluation of retrieval performance for this study was conducted using multiple indicators, including retrieval count, relevance score distributions, query response time, and retrieval-specific metrics such as Precision@k and Recall@k. The retrieval count was configured to return seven documents per query, serving as a consistency check for the pipeline. Any deviation from this expected count may indicate inefficiencies or misconfigurations in the retrieval mechanism. The relevance scores, generated by the ChromaDB vector store’s embedding function, measure the degree of similarity between queries and retrieved documents. A distribution skewed toward higher scores reflects stronger retrieval quality, whereas irregularities or lower values may suggest limitations in the embedding model or retrieval process. Furthermore, query response time serves as a key indicator of system efficiency, directly influencing the quality of real-time user interaction. Lower response times indicate smoother performance and enhanced usability, often corresponding to simpler queries that require minimal retrieval or reasoning. In contrast, longer delays may result from more complex or multifaceted queries, increased retrieval depth, or intermittent connection issues, all of which can impact system responsiveness. To evaluate response times across all three languages, ten representative queries were used for each of Amharic, Afaan Oromo, and English. This ensured consistency, comparability, and a reliable assessment of system performance under multilingual conditions. To further strengthen the evaluation of the RAG component, retrieval-specific metrics, precision, and recall at *k* values were computed using a labeled benchmark that maps user queries to relevant document chunks. As *k* value increases, Recall improves consistently, indicating better coverage of relevant information as more candidate documents are retrieved. In contrast, Precision shows a slight decline before stabilizing, suggesting that additional retrieved chunks may introduce marginally less relevant results while maintaining overall relevance. This trade-off highlights the importance of selecting an appropriate k value, with moderate values providing a balanced compromise that supports effective and reliable downstream response generation. The combined results of these evaluations are summarized in Fig. 3, which illustrates the retrieval counts (Fig. 3a) where the *x*-axis represents each query (Q1, Q2, …) and the *y*-axis indicates the number of documents retrieved per query; relevance score distribution (Fig. 3b) with the *x*-axis representing scores from 0 to 1 and the *y*-axis showing the frequency of documents within each score range; and precision and recall at *k* (Fig. 3c) where *x*-axis shows top-*k* values and *y*-axis indicates scores from 0 to 1 and response times across languages (Fig. 3d) where the *x*-axis denotes the queries (Q1, Q2, …) and the *y*-axis represents the response time in seconds. The complete list of queries used for this evaluation in all three languages is provided in Supplementary Material 1.

### Grounded response results of retrieval-augmented generation

**Fig. 4:**
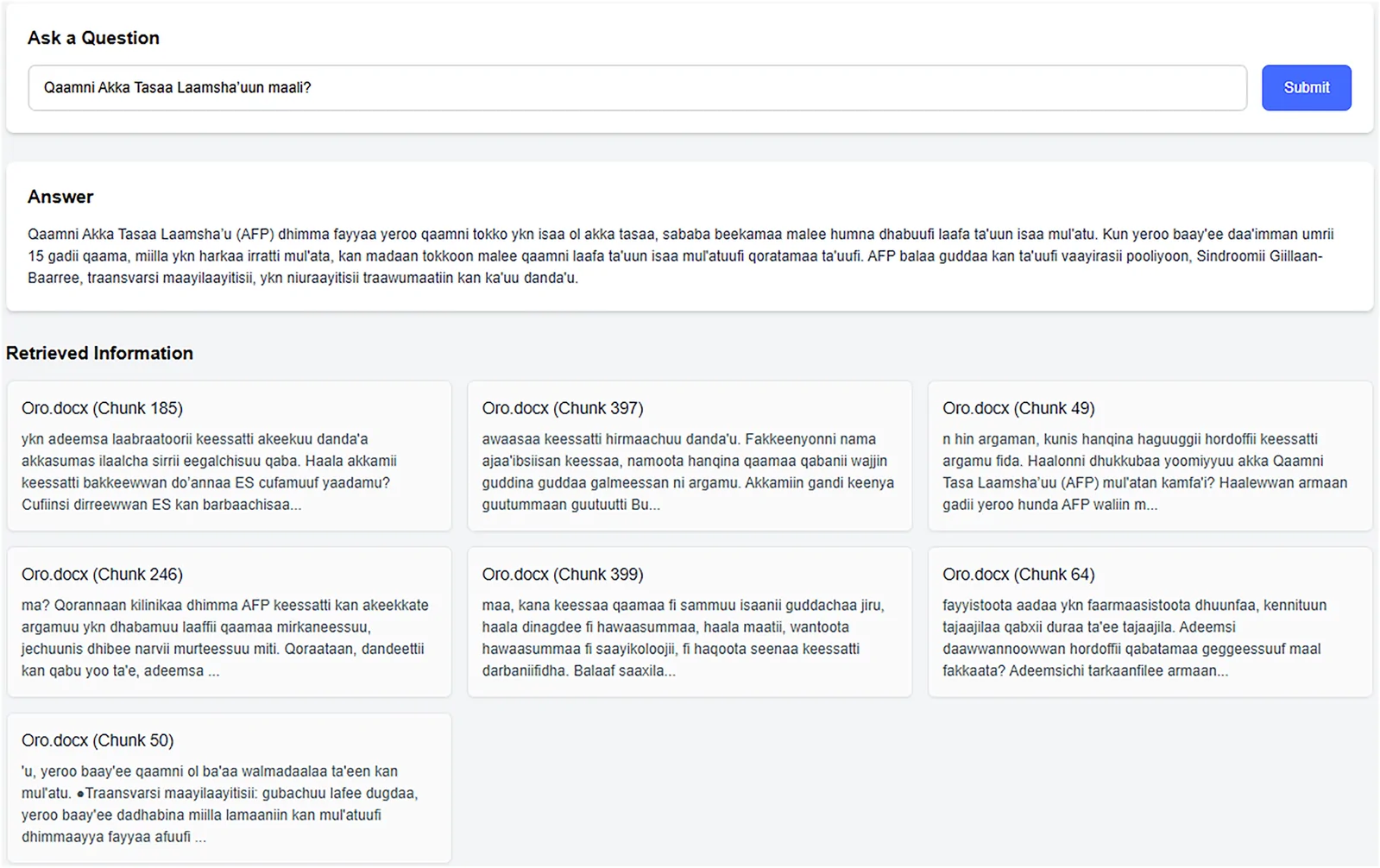
Grounded response generation for an Afaan Oromo query. The figure shows the AFP Assistant interface processing a user query in Afaan Oromo. The upper section displays the submitted query, the middle section presents the generated answer, and the lower section lists the retrieved document chunks used as supporting evidence for response generation.

**Fig. 5:**
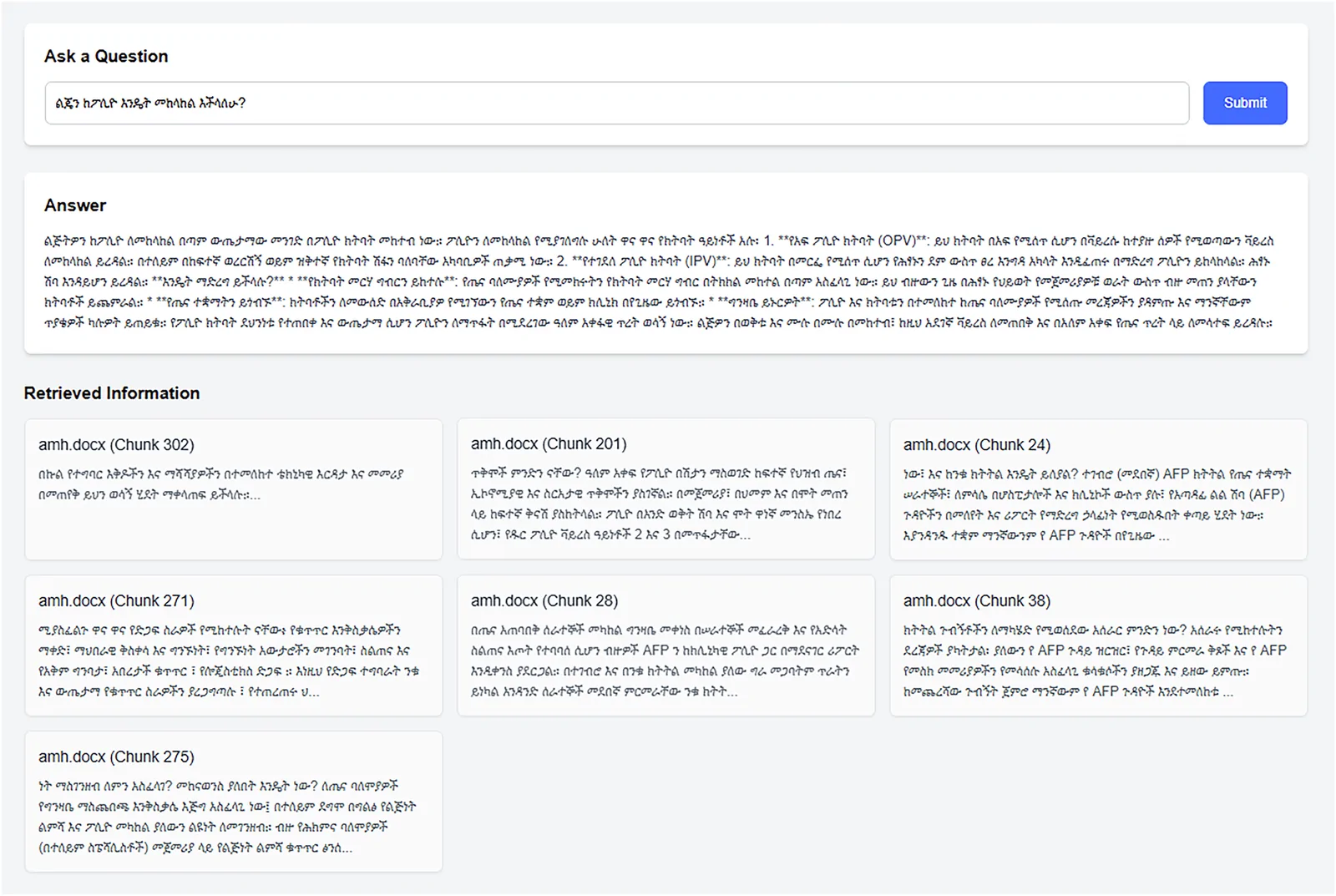
Grounded response generation for an Amharic query. The figure illustrates the AFP Assistant responding to a user query in Amharic. The query is shown at the top, the generated response is displayed in the center, and the retrieved knowledge-base chunks used to support the answer are presented below.

**Fig. 6:**
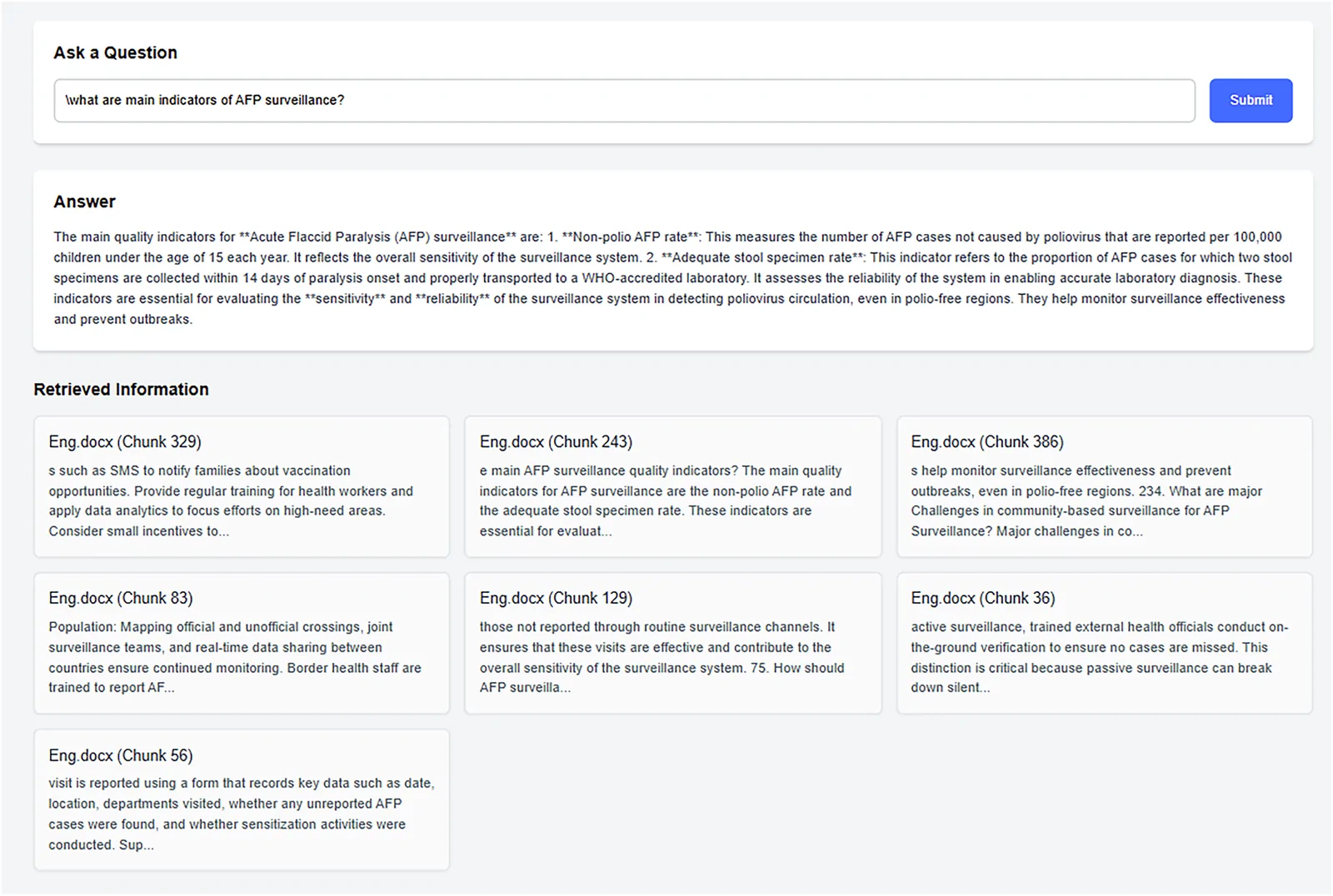
Grounded response generation for an English query. The figure demonstrates the AFP Assistant’s retrieval and response workflow for an English-language query. The interface displays the user question, the generated response, and the retrieved document segments that provide evidence for the generated answer.

The AFP Assistant’s responses were evaluated through an internal, developer-focused interface designed for testing. This interface allows detailed inspection of the model’s query processing pipeline, including document segmentation, indexing, chunk creation, and retrieval accuracy, highlighting which chunks and language sources are accessed for each query. Retrieved document segments are displayed alongside generated responses, enabling verification of evidence and enhancing grounding accuracy, transparency, and trust, critical aspects for health applications. The evaluation focused on two main dimensions: answer quality (correctness, completeness, and clarity) and retrieval quality (relevance of supporting evidence). Figures 4–6 demonstrate that the system consistently grounds responses in accurate, language-specific sources for Afaan Oromo, Amharic, and English queries, respectively.

### System evaluation results

The following section presents the evaluation results and corresponding analysis based on the held-out test set, focusing on system performance across languages and representative task categories. As summarized in Table 1, the system demonstrates consistently strong performance across all languages, with slightly higher scores observed for English, while maintaining robust performance in low-resource languages. Across task categories, the model effectively handles both symptom-related and informational queries, indicating its suitability for supporting diverse CBS interactions. These results provide a realistic estimate of the system’s generalization capability, as they are derived from data not seen during development or tuning.

**Table 1:** Stratified performance across languages and different tasks

Task	Amharic (Score)	Afaan Oromo (Score)	English (Score)	Combined average
AFP symptom	0.88	0.86	0.91	0.88
Vaccination information	0.92	0.89	0.94	0.91
Case reporting queries	0.85	0.84	0.89	0.86
General health advice	0.90	0.88	0.93	0.90
Total average	0.89	0.87	0.92	0.89

Findings from the user-centered evaluation indicate that the system is practical and contextually appropriate for deployment in real-world healthcare settings. The feedback consistently emphasized the importance of multilingual support, particularly in Afaan Oromo and Amharic, as it improved comprehension, usability, and user trust. This capability was especially valuable for frontline health workers and community members, enabling more effective communication and dissemination of health information. For example, a community health worker at Shenen Gibe General Hospital (ShGGH) reported that the AFP Assistant was easy to integrate into routine activities and helped deliver clear health messages to patients in their preferred language, thereby improving engagement during community interactions. Although minor errors were reported, the overall findings indicate that the system is suitable for deployment in low-resource settings.

In addition to the overall evaluation, an error analysis was conducted during in-house (developer) testing to identify recurring limitations in chatbot responses across supported languages. Several common failure modes were observed, primarily related to language consistency, handling of short queries, and occasional inaccuracies in response generation despite correct language detection. Table 2 summarizes these failure modes with representative examples.

**Table 2:** Common failure modes identified during in-house evaluation of the AFP Assistant across Amharic, Afaan Oromo, and English, including representative queries, observed issues, and potential causes

Error type	Example query	Observed issue	Possible cause
Responding not in the user query language		The system responded correctly in English instead of Amharic (the query language)	Language detection or response generation mismatch
Short Amharic query not recognized		The system failed to retrieve relevant information	Very short queries provide insufficient semantic context for retrieval
Incorrect response despite correct language detection		Response generated but contained partially incorrect information	Retrieval mismatch or limited contextual grounding
Too short responses		Response provided correct information but lacked sufficient explanation	Response generation settings favor concise outputs
Language not detected, and wrong response		An incorrect or irrelevant response was generated	Failure in the language detection pipeline for noisy or ambiguous input

Many of the identified errors were progressively mitigated through iterative system refinement, including improvements to the language detection pipeline, retrieval logic, preprocessing strategies, and embedding methods. The integration of structured system instructions and retrieval prompt templates further enhanced language consistency, contextual grounding, and response completeness.

Despite these improvements, a number of residual limitations remain in the current deployed system, as observed during evaluation. These issues reflect the behavior of the chatbot under real-world usage conditions. The system occasionally exhibits challenges in language detection when handling mixed-language inputs, resulting in responses being generated in the incorrect language. Performance is also comparatively weaker for low-resource languages such as Afaan Oromo and Amharic, leading to responses that may be less detailed or less precise than those in English. In addition, response latency varies depending on retrieval complexity. Furthermore, some responses, particularly in Afaan Oromo, tend to be longer or insufficiently segmented, reflecting limitations in sentence structuring.

**Fig. 7:**
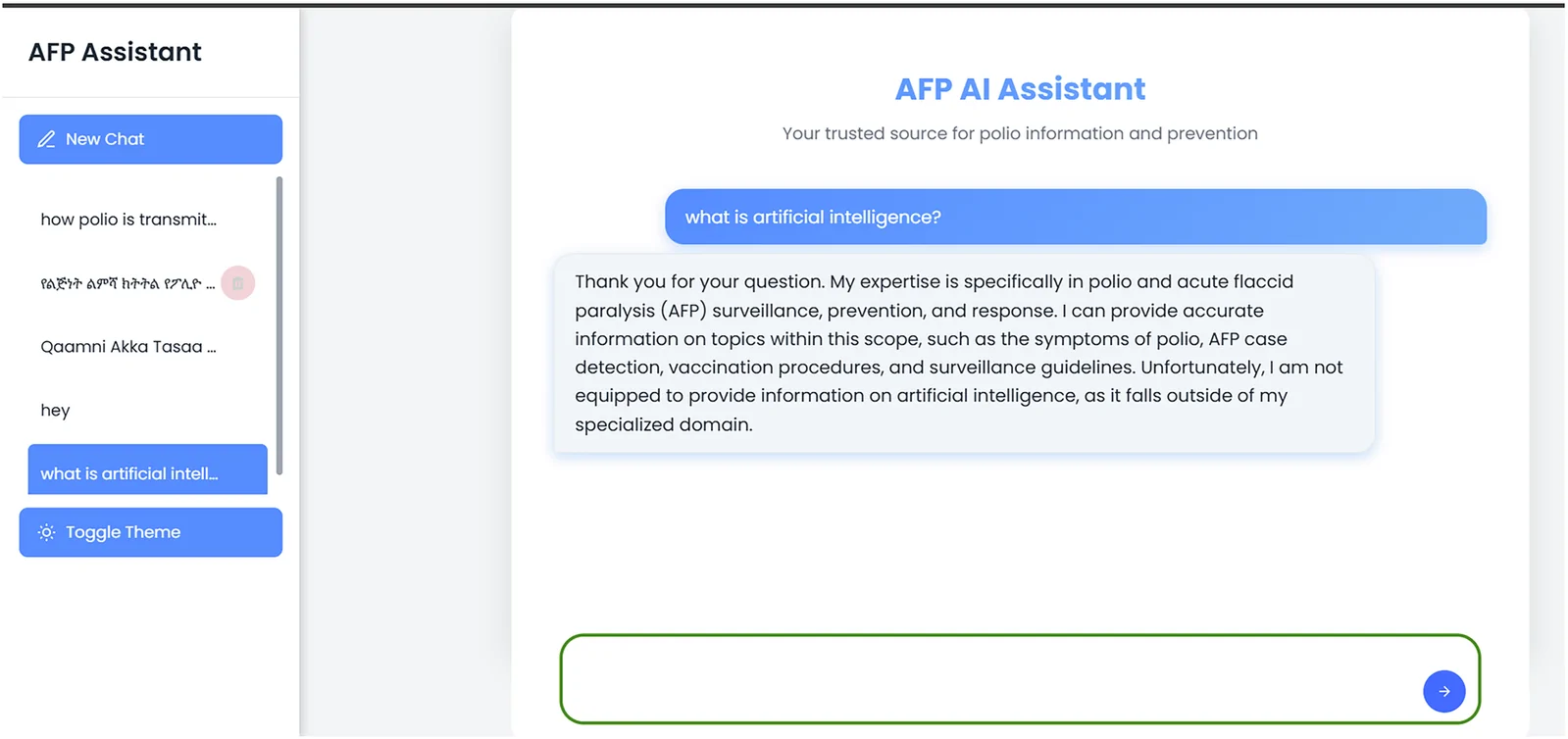
Example of chatbot refusal behavior for an out-of-scope query. The system identifies queries unrelated to polio or AFP surveillance and responds with a safe refusal.

Beyond these performance-related limitations, system safety was also explicitly evaluated to ensure reliable behavior under unexpected inputs. To assess this, the chatbot’s response to out-of-scope queries was examined. As illustrated in Fig. 7, when users submit unrelated questions, the system avoids generating misleading information and instead produces a safe refusal response, explicitly indicating that the query falls outside its scope. This mechanism reduces the risk of misinformation and is enforced through system-level instructions and domain-specific prompting that constrain responses to the curated polio knowledge base.

### Comparison with the state-of-the-art models

**Fig. 8:**
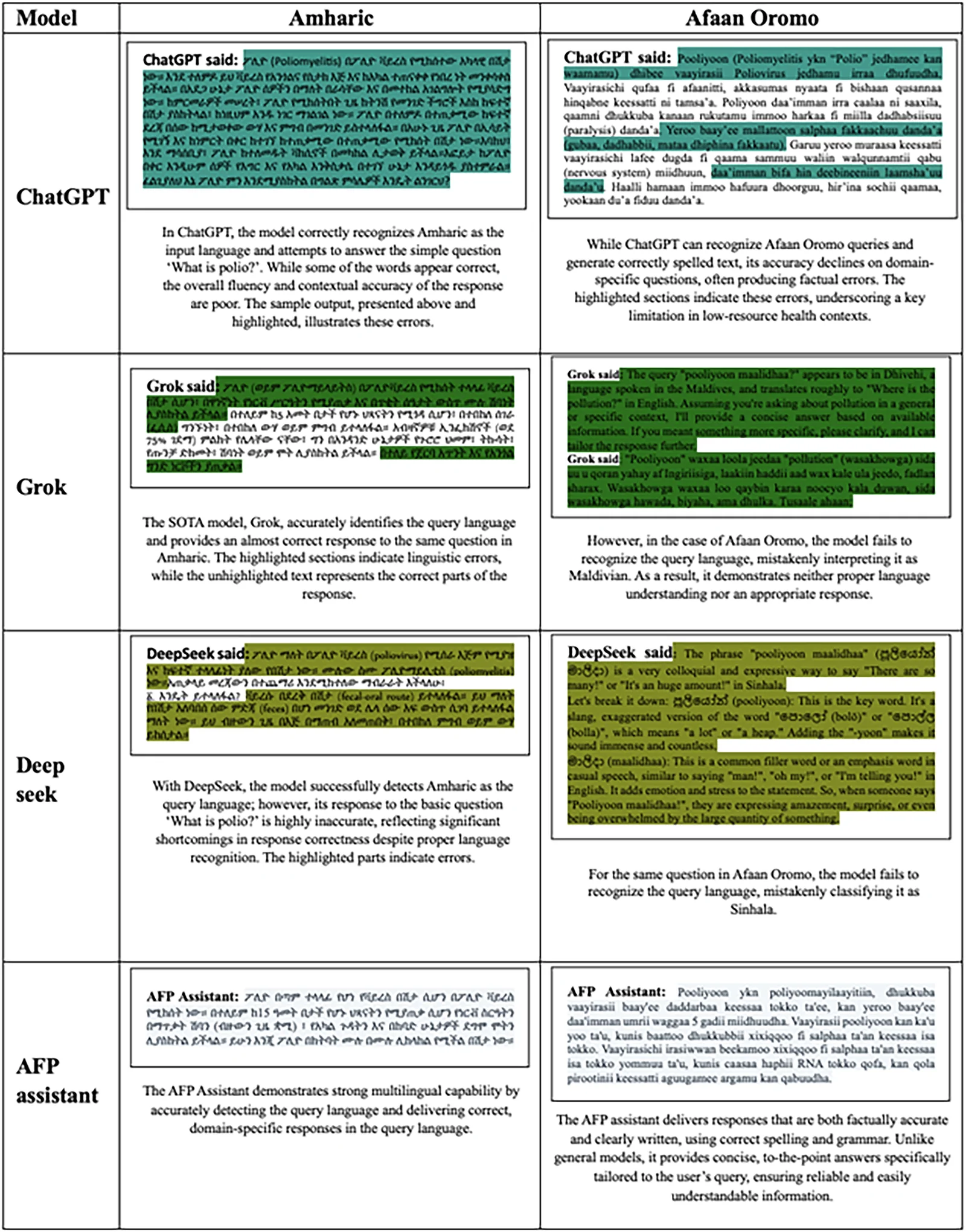
Comparison of AFP Assistant and state-of-the-art language models. Panels compare responses generated by AFP Assistant, ChatGPT, Grok, and DeepSeek for equivalent AFP-related queries in English, Amharic, and Afaan Oromo. The figure highlights differences in multilingual understanding, language consistency, contextual relevance, and domain-specific accuracy, with AFP Assistant demonstrating stronger performance in low-resource language settings.

In the comparative evaluation, the AFP Assistant was benchmarked against three SOTA LLMs, ChatGPT, Grok, and DeepSeek using a standardized set of AFP-related queries designed to reflect the practical information needs of frontline health workers. These queries were administered in English, Amharic, and Afaan Oromo to rigorously assess multilingual performance across both high- and low-resource language settings. To ensure methodological consistency and comparability, the same evaluation framework employed for system assessment was adopted for the comparative analysis. Specifically, a structured rubric (Table 4) was applied, encompassing multiple dimensions of response quality, including factual correctness, completeness, clarity and understandability, level of detail, structure and organization, contextual relevance, domain specificity, and local language response quality. All model outputs were systematically evaluated using a 5-point Likert scale (1 = very poor, 5 = excellent) across these criteria. The evaluation was conducted by six domain experts with relevant linguistic and public health expertise, who independently assessed the responses based on the predefined rubric. Final scores were obtained by aggregating expert ratings, and in cases of differing judgments, the final decision was determined through consensus among the experts. This expert-driven evaluation approach ensures a robust and fair comparison across models, while capturing critical differences in domain-specific accuracy and performance in low-resource languages. From the evaluation set, a representative query “What is polio?” posed in both Amharic and Afaan Oromo is presented in Fig. 8 to qualitatively illustrate the comparative behavior of the models.

All three SOTA models performed well when responding to simple queries about polio in English, demonstrating strong baseline capabilities in high-resource languages. However, their performance significantly declined in low-resource languages such as Amharic and Afaan Oromo. In these cases, model behavior was inconsistent: when a query was handled reasonably well in one language, performance in the other often deteriorated, with frequent failures in language detection or inaccuracies in domain-specific medical responses. In contrast, the AFP Assistant demonstrated stronger adaptability to low-resource settings. Supported by a dedicated multilingual user interface designed to handle Amharic, Afaan Oromo, and English inputs, it consistently recognized queries across these languages and produced more contextually relevant and medically accurate responses. Although occasional minor errors occurred, the system’s ability to manage multilingual interactions and deliver reliable domain-focused outputs underscores its superiority over the benchmark SOTA models in the context of polio awareness and AFP-related information delivery (see Fig. 8).

## Discussion

This study introduced the AFP Assistant, a multilingual AI-powered chatbot designed to strengthen the CBS of AFP in Ethiopia. The system addresses critical barriers to effective surveillance, linguistic and informational inequity by providing reliable, culturally appropriate polio-related information in Amharic, Afaan Oromo, and English. Unlike most existing health chatbots, which primarily target resource-rich languages, the AFP Assistant explicitly prioritizes low-resource language contexts, filling a gap in polio surveillance and awareness.

The results demonstrate that the hybrid architecture of SFT combined with RAG effectively improves accuracy, contextual grounding, and multilingual robustness. In particular, the findings highlight the effectiveness of transfer learning and multilingual modeling for low-resource languages such as Amharic and Afaan Oromo. By leveraging a pretrained multilingual model, the system is able to transfer rich linguistic and semantic knowledge from high-resource languages, significantly improving performance despite limited labeled data. The use of a parallel multilingual corpus further supports cross-lingual alignment, enabling the model to benefit from English-language supervision while maintaining semantic consistency across languages. Additionally, the integration of RAG plays a critical role in improving factual grounding by injecting contextually relevant knowledge during inference, thereby reducing hallucinations. The adoption of PEFT within the Vertex AI environment further demonstrates that effective domain adaptation can be achieved with limited computational resources, making the approach practical for real-world deployment in low-resource settings. Finally, Gemini 2.5-flash was identified as the most suitable model due to its superior handling of low-resource languages, outperforming other SOTA LLMs in both RC and LU.

These findings have significant implications for Ethiopia’s surveillance system. By empowering communities to recognize symptoms, report suspected AFP cases, and access prevention guidance in their own languages, the AFP Assistant enhances both the sensitivity and timeliness of CBS. It also supports health workers and volunteers with on-demand resources, thereby strengthening local capacity and bridging critical gaps in communication and trust, particularly in rural and underserved areas where conventional health communication tools often fall short.

A few limitations should be acknowledged. The scarcity of structured datasets in Amharic and Afaan Oromo required extensive translation and validation by native speakers and health experts, a resource-intensive process that may have introduced subtle biases. Computational limitations restricted large-scale experimentation, as even smaller LLMs demanded significant GPU resources, and the cost of fine-tuning limited the use of larger adapters for parameter-efficient training. Evaluation relied primarily on human judgment through query testing, a common practice in generative model research but inherently subjective, especially in the absence of standardized benchmarks for Amharic and Afaan Oromo. Technical challenges arose during preprocessing and data handling, particularly with large JSONL text entries; token limits sometimes caused encoding errors, memory overloads, or data truncation, necessitating careful chunking and strict validation. Furthermore, the system currently depends on stable internet connectivity, which may limit accessibility in the most resource-constrained communities.

Despite these challenges, the AFP Assistant demonstrates the feasibility and value of AI-driven, multilingual health tools in low-resource settings, providing a practical pathway for strengthening CBS. Its modular architecture enables adaptation to other public health contexts by updating the knowledge base and incorporating disease-specific datasets and guidelines without requiring major changes to the core model. The approach can be adapted to high-burden and emerging diseases, such as measles, malaria, tuberculosis, and Mpox, by integrating relevant clinical and surveillance information. In addition, the framework is designed to scale to other low-resource language settings through the use of multilingual models and RAG, enabling accurate handling of linguistically diverse medical content. While careful selection of models and retrieval components remains important, the overall system is highly adaptable. Future work includes expanding language coverage (e.g., Somali and Afar), improving offline and low-bandwidth functionality, integrating with national health information systems for real-time reporting, and supporting structured data collection during community visits. Collectively, this approach has the potential to evolve into a multilingual, culturally adapted, and community-centered AI platform that supports disease prevention, surveillance, and public health resilience across Africa.

## Methods

### AFP Assistant overview

The AFP Assistant is built on a hybrid architecture that combines fine-tuning LLMs with a RAG framework. Fine-tuning embeds domain-specific knowledge, while RAG enables accurate, context-aware access, and up-to-date responses drawn from a structured knowledge base. To ensure inclusivity, the system incorporates advanced NLP techniques with multilingual support for low-resource languages such as Amharic and Afaan Oromo, enabling equitable access to health information across diverse communities.

**Fig. 9:**
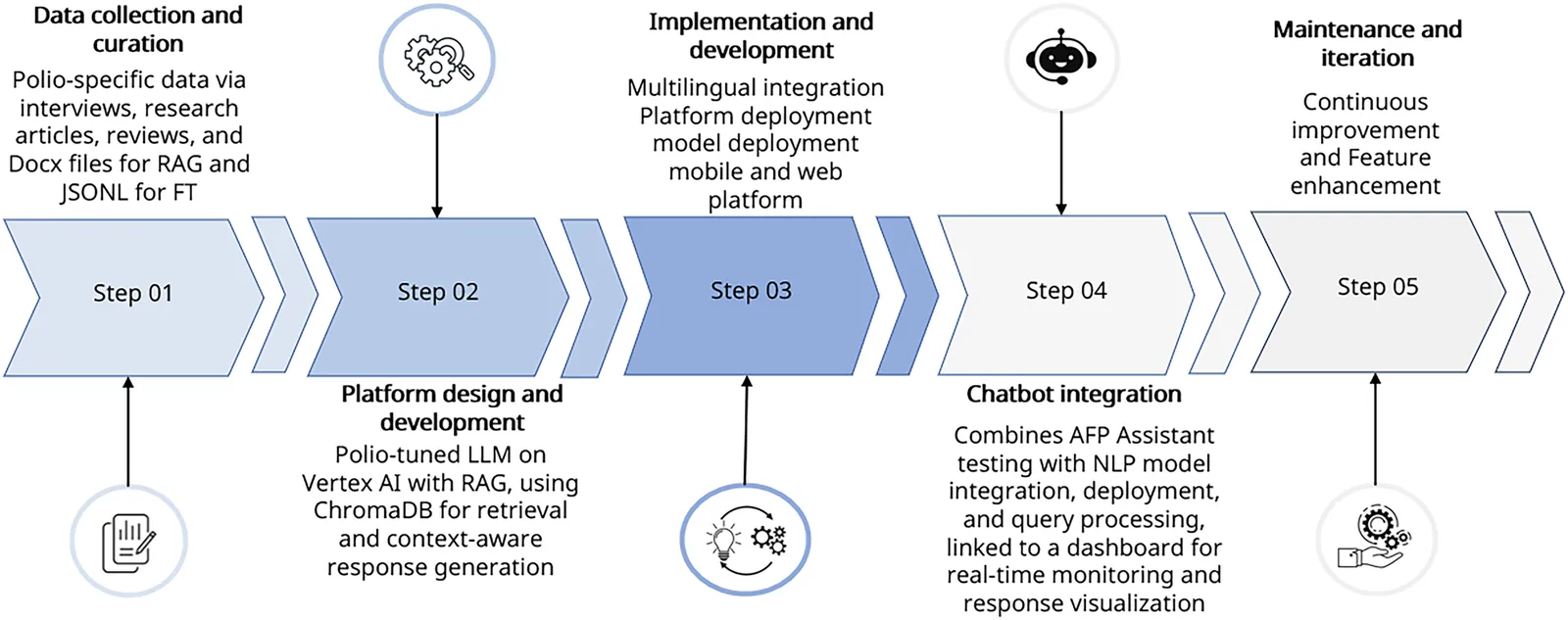
Overview of the AFP Assistant development process. The five-step framework illustrates the progression from data collection and curation (Step 1), platform design and development (Step 2), implementation and deployment (Step 3), chatbot integration and testing (Step 4), and ongoing maintenance and iteration (Step 5). Each step highlights key activities, including polio-specific data collection and preparation, LLM fine-tuning with RAG, platform deployment, NLP integration, real-time monitoring, and continuous feature enhancement.

AFP Assistant was developed in a structured five-step process. It began with data collection and curation, where polio-specific datasets were obtained from interviews, research articles, reviews, and documents. These were formatted into RAG-compatible files for retrieval and JavaScript object notation lines (JSONL) files for fine-tuning, ensuring the models are trained on reliable and relevant data. The platform design and development phase involved fine-tuning selected LLMs on Vertex AI^56^, integrating them with a RAG framework and ChromaDB^57^ for efficient retrieval and context-aware response generation. The fine-tuned model is then deployed to a Vertex AI endpoint, forming the system’s technical backbone. The implementation and development phase focused on multilingual integration and deployment across mobile and web platforms to ensure accessibility and scalability. In the chatbot integration stage, the AFP Assistant was tested and integrated with the fine-tuned NLP model for effective query processing and real-time response generation. A live monitoring dashboard enabled visualization of user interactions and system performance, supporting real-time evaluation and decision-making. Finally, the maintenance and iteration phase ensured continuous improvement and feature enhancement through regular updates and user feedback, strengthening the chatbot’s accuracy, responsiveness, and long-term reliability in supporting polio surveillance and public health communication in Ethiopia. An overview of the methodology is presented in Fig. 9.

### System architecture

**Fig. 10:**
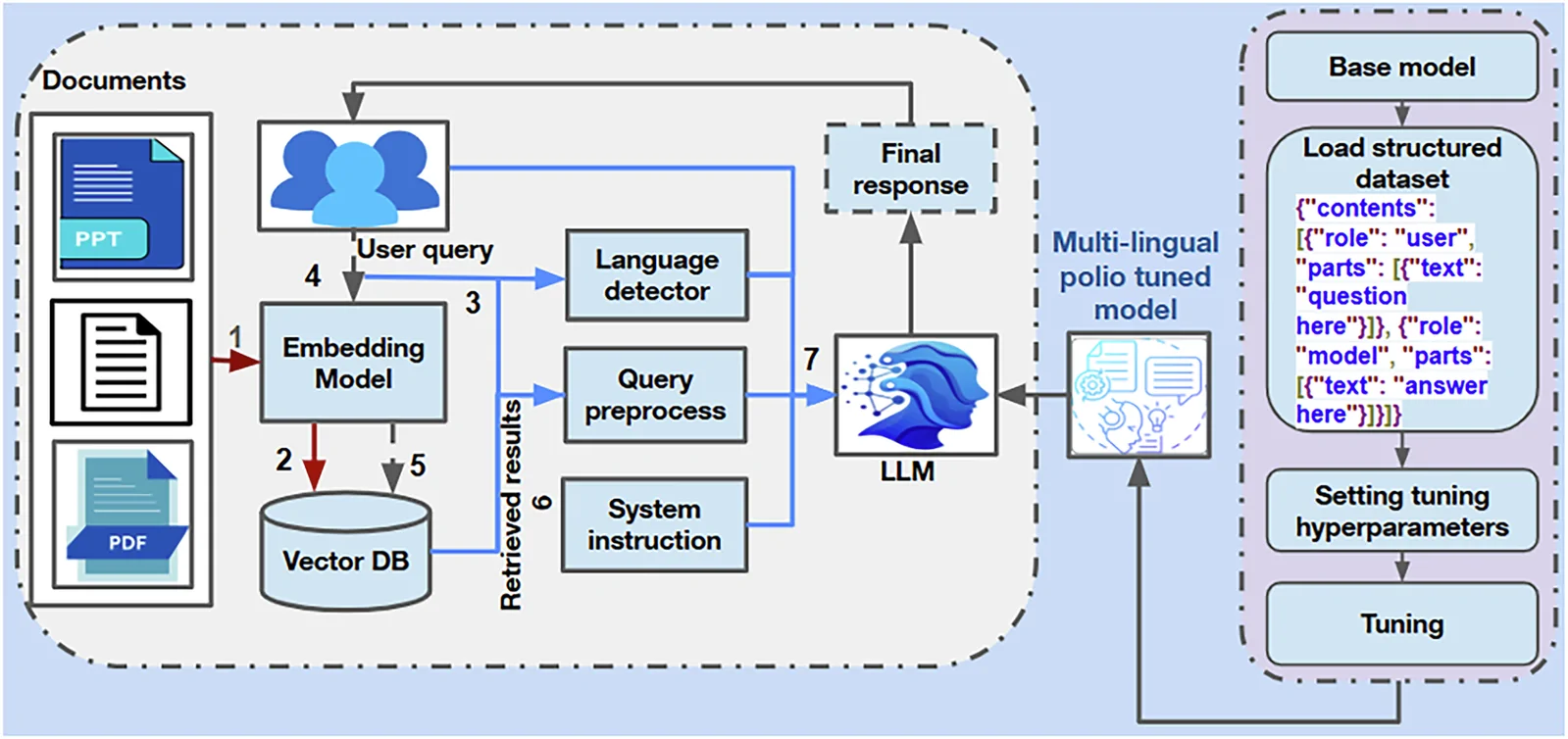
System architecture showing three layers, namely data ingestion and storage, query processing and retrieval, and response generation. Documents are embedded and stored in a vector database. User queries are processed through an embedding model, language detector, and query preprocessor to identify language and context. Retrieved results, combined with system instructions, are passed to a fine-tuned multilingual LLM trained on structured datasets to generate the final response.

The AFP Assistant system architecture is organized into three main layers: data ingestion and storage, query processing and retrieval, and response generation. In the data ingestion and storage layer, relevant data sources, including medical guidelines, research articles, and community insights, are ingested, preprocessed, and stored in a structured knowledge base. The query processing and retrieval layer manages user interactions by first detecting the input language, specifically distinguishing among Amharic, Afaan Oromo, and English, to route queries appropriately. Once identified, the query is embedded into a vector representation and used to retrieve semantically relevant text segments from the knowledge base via a vector database. In the response generation layer, the retrieved evidence is combined with the user’s query and passed to the fine-tuned LLM, which generates outputs that are both medically accurate and culturally appropriate. This multilingual and hybrid design ensures synergy between the two components, with fine-tuning that embeds stable domain knowledge on polio, while RAG enables dynamic access to updated information, together reducing hallucinations, improving cross-lingual consistency, and enhancing factual reliability. Figure 10 illustrates the key components of the system and the flow of information.

### Datasets collection and curation

Comprehensive textual data related to poliomyelitis, covering symptoms, causes, prevention, diagnosis, treatment, and surveillance, were collected through a systematic and reproducible protocol. The data sources included peer-reviewed research and review articles retrieved from academic databases such as Google Scholar, PubMed, Scopus, Web of Science, ScienceDirect, JSTOR, DOAJ, IEEE Xplore, and ResearchGate, as well as authoritative guidelines, policy documents, and educational materials obtained from reputable websites, including the World Health Organization, the GPEI, the Ethiopian Ministry of Health (MoH), the Ethiopian Public Health Institute, the Oromia Health Bureau, the Centers for Disease Control and Prevention, and the Mayo Clinic. Database searches were conducted using structured keyword combinations polio, poliomyelitis, AFP, surveillance, epidemiology, vaccination, and community health, with queries adapted to each platform and restricted to English-language publications from 2002 to 2024. Importantly, the record counts reported apply only to research and review articles retrieved from academic databases, while website-based materials were used to supplement and contextualize the dataset. The search yielded 260 records, of which 36 duplicates were removed, leaving 224 records for screening; 94 records were excluded based on date and relevance. Following a full-text assessment of 130 articles, 70 were excluded due to limited relevance, outdated guidance, redundancy, or the presence of subjective, non-evidence-based claims, including repeated textual segments across sources. This process resulted in a final dataset of 60 high-quality research and review articles, complemented by authoritative web-based resources to ensure accuracy, completeness, and contextual consistency.

Additional insights were collected through interviews, employing purposive sampling to engage 15 respondents with direct experience in Ethiopia’s AFP surveillance system, including healthcare professionals, surveillance officers, and CVs. The use of purposive sampling enhanced the contextual relevance and validity of the findings, ensuring that user-centered perspectives informed both the assessment of system readiness and the development of the multilingual chatbot. The interviews served to gather insights that could be directly integrated into the design of the AFP Assistant, ensuring that its content and features addressed real community needs. The interview material is provided in Supplementary Material 2.

Since most available resources were in English, the entire dataset was carefully translated into both local languages (Amharic and Afaan Oromo). To ensure accuracy and cultural relevance, translations underwent rigorous review by language experts, with native speakers and local medical professionals contributing to the translation, transcription, and verification of medical terminology. A parallel corpus was then developed by aligning English sentences with their Amharic and Afaan Oromo equivalents, ensuring linguistic consistency while preserving technical precision.

**Fig. 11:**
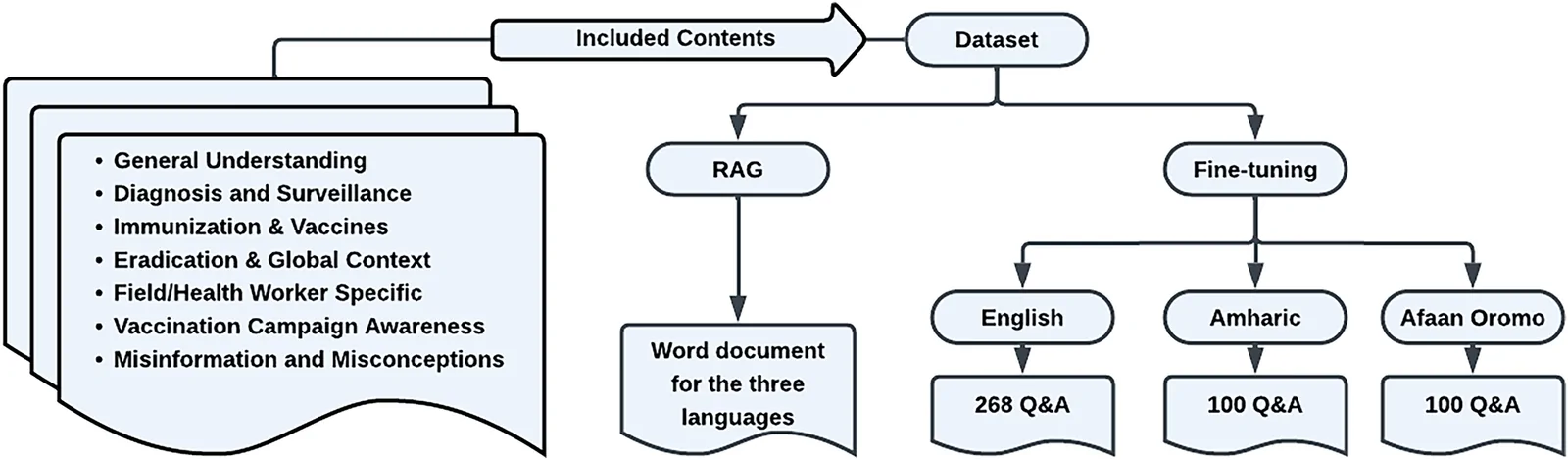
Overview of dataset contents collected, curated, and distributed for this study. RAG retrieval augmented generation, Q&A question and answers.

To support both the retrieval and fine-tuning processes, the dataset consisted of two formats: a document corpus for RAG and a structured question–answer dataset for LLM fine-tuning. The RAG knowledge base was constructed from curated health resources, comprising approximately 215 pages in English, 172 pages in Amharic, and 222 pages in Afaan Oromo. For supervised fine-tuning (SFT), a structured JSONL dataset of question–answer pairs was developed, including 268 pairs in English, 100 in Amharic, and 100 in Afaan Oromo. To enhance contextual relevance, around 50 pairs in each of the local languages were obtained from primary data collection, reflecting locally expressed questions and community-level information needs. These were compiled using locally produced materials such as flyers, case definitions, and training resources developed by the MoH, in addition to interviews. Overall, for Amharic and Afaan Oromo, approximately 18.6% of the dataset was derived from primary sources, while the remaining data was obtained through translation. This reflects the limited availability of publicly accessible health information in these languages and underscores the importance of incorporating locally generated content to improve linguistic and contextual relevance. This comprehensive multilingual integration of both primary and translated data provides a robust foundation for the development of the AFP Assistant, enhancing inclusivity, linguistic relevance, and accessibility for Ethiopia’s diverse communities. Figure 11 presents an overview of the dataset preparation and classification workflow for RAG and model fine-tuning. As illustrated, source materials were systematically collected across key thematic domains, including general understanding, diagnosis and surveillance, immunization and vaccines, eradication and global context, field and health worker specific content, vaccination campaign awareness, and misconceptions.

### Large language model selection

Selecting an LLM is a critical step in developing multilingual healthcare applications. For this work, the chosen models needed to provide robust multilingual support, particularly for low-resource languages such as Amharic and Afaan Oromo, while also being adaptable for domain-specific fine-tuning on polio. Model selection was guided by a strategy that considered both open-weight and managed API-based approaches. First, open-weight models, including Meta’s LLaMA 3.1, Google’s Gemma 3, and DeepSeek-R1, were considered based on their accessibility for fine-tuning, compatibility with multilingual tasks, and flexibility for customization. In parallel, a managed API-based model (Google’s Gemini 2.5-flash) was also included to address limitations observed with open-weight approaches and to provide a comparative perspective. This approach enabled a balanced evaluation of customization capabilities alongside performance and deployment considerations. Suitability was evaluated through query-based tests in English, Amharic, and Afaan Oromo, using five benchmark questions per model (queries are provided in Supplementary Material 3). Performance was assessed based on each model’s ability to interpret queries, generate accurate responses in the appropriate language, and demonstrate awareness of polio-related content. Additional evaluation criteria included accessibility and licensing, compatibility with fine-tuning pipelines, pretraining coverage across multilingual corpora, and efficiency in resource-constrained environments.

As summarized in Table 3, several state-of-the-art (SOTA) open-source models, including LLaMA 3.1, Gemma 3, and DeepSeek-R1, offer valuable capabilities but present limitations that affect their suitability for this research. LLaMA 3^58^ provides open weights and supports both full and parameter-efficient fine-tuning (PEFT) methods, such as low rank adaptation (LoRA) and quantized low rank adaptation (QLoRA). However, its language coverage does not explicitly include low-resource languages such as Amharic and Afaan Oromo, and its larger variants demand extensive graphical processing unit (GPU) memory, making it impractical for locally constrained computational setups. Similarly, Gemma 3^59^ is fully open-source, fully tunable, and supports over 140 languages, making it a strong candidate for multilingual applications. Yet, despite its efficiency relative to other large models, Gemma 3 still requires substantial computational resources for effective fine-tuning, which exceed the capacity of the available local infrastructure. DeepSeek-R1^60^, although notable for its advanced reasoning abilities and cost efficiency, primarily supports English and Chinese, exhibiting weak performance in African low-resource contexts. Its extremely large model size and dependence on multi-GPU further limit its practicality for this study. In contrast, Google’s Gemini^61^ demonstrated clear advantages across multilingual capability, adaptability, and deployment scalability. The Gemini 2.5-flash model, accessed through Vertex AI, explicitly supports Amharic and other low-resource languages and integrates seamlessly with RAG pipelines via managed application programming interfaces (APIs). Although not open-source, its selection was driven by practical considerations, including limited local computational resources, the need for reliable multilingual performance, and ease of deployment in real-world healthcare settings.

**Table 3:** Language model comparison based on criteria

Criteria	Multilingual	Open source	Fine-tuning adaptability	Model size and efficiency	Reference
Gemini 2.5-flash (Google)	Supports 100+ (explicitly supports Amharic)	Managed via Vertex AI	(supervised tuning with adapters)	Input size limit is 500 MB; the model runs on high-compute infrastructure	^68^
Llama 3.1	Supports 8 (no explicit mention of Amharic, Afaan Oromo)	Open source	Supports full fine-tuning and PEFT tuning	Up to 405B parameters require ~810 GB VRAM at FP16; the 70B needs ~140 GB	^69,70^
Gemma 3	Supports 140+ languages	Open source	Fully tunable	Parameter sizes are 270 M, 1B, 4B, 12B, and 27B; expensive to run in terms of memory cost and power consumption	^71,72^
DeepSeek-R1	English and Chinese, no explicit coverage of Amharic and Afaan Oromo	Open source	Fully tunable	It is 671B total (37B active weights); requires large multi-GPU setups	^73–75^

In addition to optimizing model performance, the selection of the tuning and deployment environment was driven by considerations of reliability, scalability, and secure system management, critical factors in healthcare contexts. Leveraging Vertex AI offers a fully managed platform for fine-tuning and deployment, enabling controlled training workflows, robust performance monitoring, and reproducible results. The platform also ensures secure data handling and seamless integration with backend services, which is essential for deploying AI-driven health applications in resource-constrained settings. By offloading infrastructure management to a managed environment, Vertex AI reduces operational overhead while maintaining dependable and scalable system performance.

### Dataset preparation for fine-tuning on Gemini

**Fig. 12:**
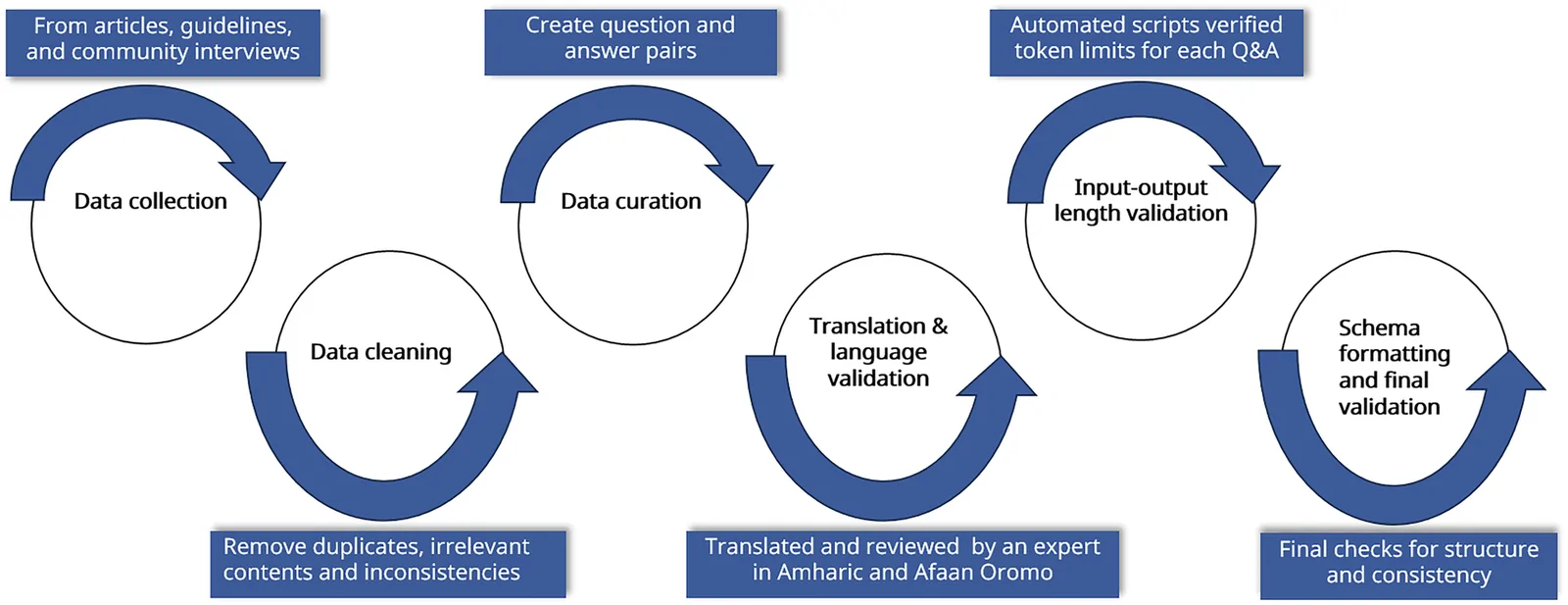
Workflow of dataset preparation showing sequential steps from data collection to final validation across three languages, including token-length verification for fine-tuning compatibility.

After data collection, the dataset underwent comprehensive preprocessing to ensure quality and consistency. Initially, all question–answer pairs were manually developed in English. These entries were then translated and culturally adapted into Amharic and Afaan Oromo by language experts, with native speakers and medical professionals verifying terminology and contextual accuracy. The translated data were aligned to form a parallel multilingual corpus across the three languages. Subsequently, duplicate entries, irrelevant content, and formatting inconsistencies were removed, and the curated data were organized into high-quality question–answer pairs. Each pair was tokenized to meet input–output length constraints (up to 5000 tokens per sample), structured into role-based formats, and converted into JSONL format for compatibility with Gemini’s SFT pipeline. Strict validation procedures ensured that each entry adhered to the defined schema, with nonconforming samples discarded to maintain data reliability and structural integrity. These preprocessing and quality assurance steps were particularly critical for low-resource languages, where data precision directly affects model accuracy and fine-tuning performance. The overall dataset preparation workflow is illustrated in Fig. 12.

### Supervised fine-tuning of Gemini 2.5-flash for domain adaptation

SFT is a targeted training approach that adapts a pre-trained LLM to domain-specific tasks using a carefully curated dataset of question–answer pairs or task-oriented examples^62^. This process adjusts the model’s parameters to internalize specialized knowledge, terminology, stylistic nuances, and reasoning patterns relevant to the target domain. In healthcare, SFT has been effectively applied to tasks such as diagnosis support, clinical note summarization, and medical entity recognition. Its advantages include improved domain accuracy, reduced hallucinations, and lower inference latency, since relevant knowledge is embedded directly into the model. Compared to training an LLM from scratch, SFT is more computationally efficient; however, it requires high-quality labeled datasets, which are costly and time-intensive to prepare.

**Fig. 13:**
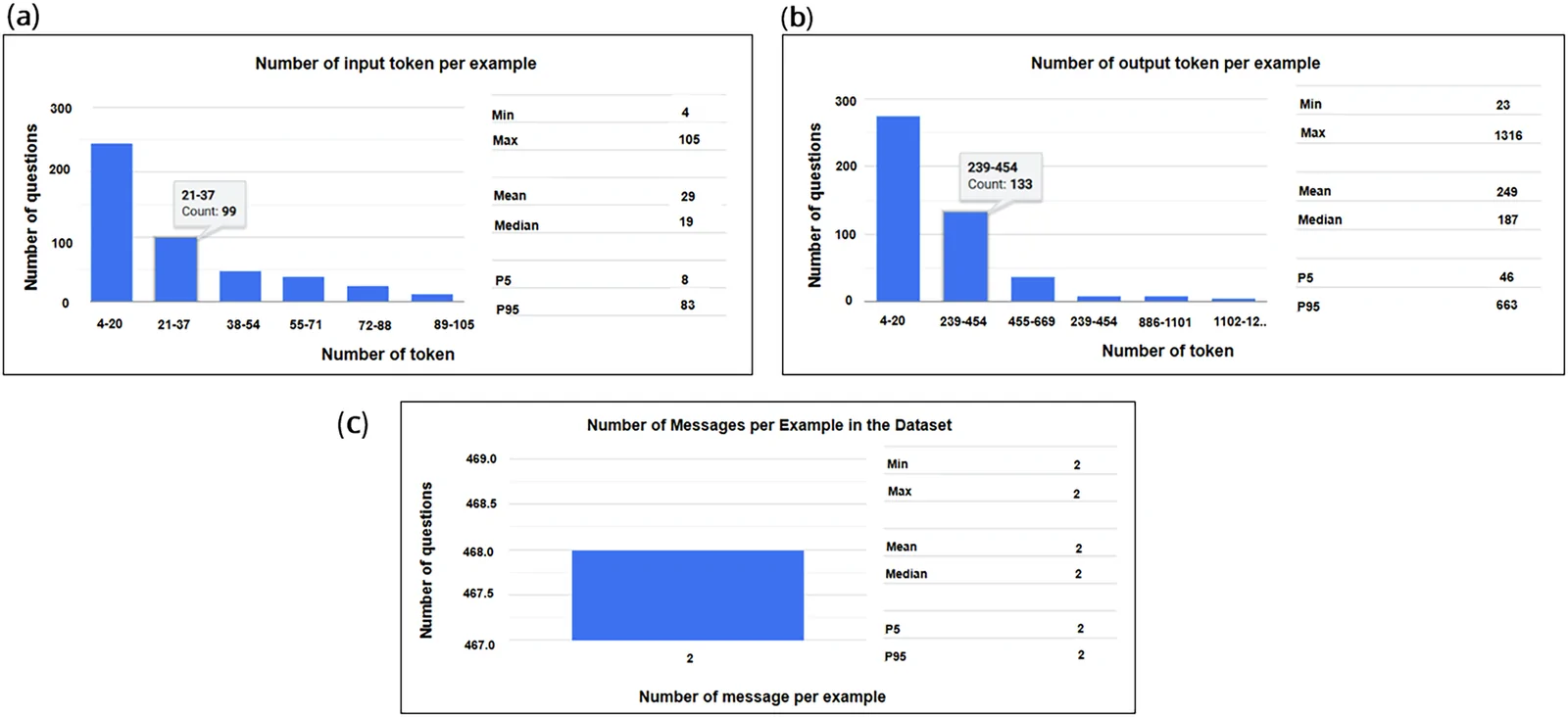
Distribution statistics of tokens and messages in the dataset. **a** Illustrates the number of input tokens per example, which range from 4 to 105, with a mean of 29 and a median of 19. **b** Demonstrates the number of output tokens per example, ranging from 23 to 1316, with a mean of 249 and a median of 187. **c** Presents the number of messages per example, indicating a consistent count of two messages across all dataset examples.

In this work, SFT was applied to Google’s Gemini 2.5-flash model using Google Cloud’s Vertex AI, with model inference performed in the us-central1 region. Vertex AI provides built-in support for fine-tuning Gemini models, enabling an efficient and scalable training pipeline. Gemini, a transformer-based generative model trained on diverse multilingual corpora, is particularly well-suited for low-resource languages, allowing the system to generate accurate and culturally appropriate responses for polio-related queries. For fine-tuning, the dataset was split into 80% training and 20% validation sets. The distribution of input–output tokens in the SFT dataset, which directly informs the tuning process, is presented in Fig. 13. To enable efficient adaptation, Vertex AI employs PEFT through adapter-based methods, where lightweight adapters are inserted into each transformer layer while the base model weights remain frozen. In this configuration, only the adapter parameters are updated, significantly reducing the number of trainable parameters and enabling training on modest computational resources.

Hyperparameter tuning was conducted to balance domain adaptation and generalization. Specifically, epochs ranging from 10 to 30 were evaluated, with performance improvements observed to plateau after 15 epochs; thus, 15 epochs were selected for the final model. A learning rate multiplier of 5 was chosen to facilitate sufficient adaptation to specialized polio and AFP knowledge while preserving general language capabilities. Additionally, an adapter size of 4 was selected to minimize the number of trainable parameters, improving efficiency and reducing the risk of overfitting. To ensure robust training, intermediate checkpoints were enabled (with a default checkpoint at epoch 10), and validation monitoring was used to detect potential overfitting. Ablation experiments against default settings further confirmed that this configuration achieved the best overall performance. Following fine-tuning, the model was deployed as a low-latency Vertex AI endpoint with a model identifier (ID: 7327333503987613696), enabling real-time, context-aware responses.

### Retrieval-augmented generation principles and implementation

RAG is a robust framework that enhances the capabilities of LLMs by integrating external retrieval mechanisms. This approach enables LLMs to produce responses that are more accurate, contextually relevant, and up-to-date by grounding outputs in a curated knowledge base^63^. In a typical RAG workflow, a user query is converted into a vector representation, which a retriever uses to search a vector database or other external sources for semantically relevant text segments. These retrieved segments augment the original query, allowing the LLM to generate responses with improved factual accuracy and contextual alignment. RAG is especially valuable in knowledge-intensive and rapidly evolving fields such as healthcare, where it reduces hallucinations, provides traceable citations, and allows knowledge bases to be updated without retraining the entire model, making it scalable and adaptable.

**Fig. 14:**
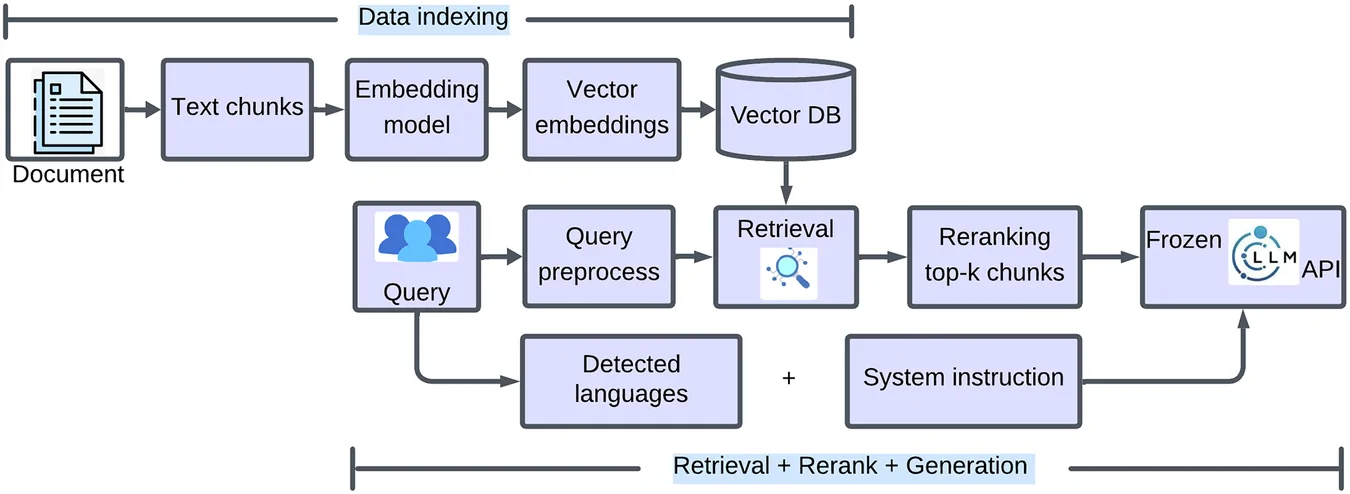
Overview of the RAG pipeline. The system first indexes documents by splitting them into text chunks, generating vector embeddings via an embedding model, and storing them in a vector database. When a user query is received, it is preprocessed and matched against stored embeddings to retrieve relevant chunks. Retrieved results are reranked, combined with system instructions, and passed to a frozen language model API for response generation.

In this study, the RAG pipeline is applied using the collected polio dataset in Amharic, English, and Afaan Oromo as the knowledge base. To support multilingual queries, the dataset in all three languages was prepared and indexed, ensuring accurate and contextually relevant chatbot interactions. Although the system can ingest multiple document formats, such as PDF, Word, and PowerPoint, this research used Word documents. After ingestion, the text from these documents was extracted and divided into multiple chunks, where each chunk contained 500 tokens. To preserve semantic continuity between the chunks, a 25-token overlap was applied across all segments, and a batch size of 100. Each chunk was then embedded using the multi-qa-mpnet-base-dot-v1^64^ sentence transformer and stored in ChromaDB along with relevant metadata for efficient retrieval. The system employs the lightweight embedding model to support English, Amharic, and Afaan Oromo queries. Although the model is primarily optimized for English, automatic language detection and language-filtered retrieval are applied to mitigate multilingual limitations. This design choice ensures effective performance under limited computational resources. As illustrated in Fig. 14, which presents the RAG workflow for this study, the system first detects the language of the user query, retrieves the most relevant chunks, reranks them, and incorporates the top results into the prompt for the polio-tuned model. A similarity-based reranking approach was used based on cosine similarity with a Hierarchical Navigable Small World index. The reranking parameters included a cosine distance metric, a top-k value of 7, and the embedding model. This choice was driven by the small dataset size, strict low-latency requirements, and limited computational resources. This process enables the tuned model to generate fact-based responses related to polio, thereby ensuring both accuracy and effective public health communication.

**Fig. 15:**
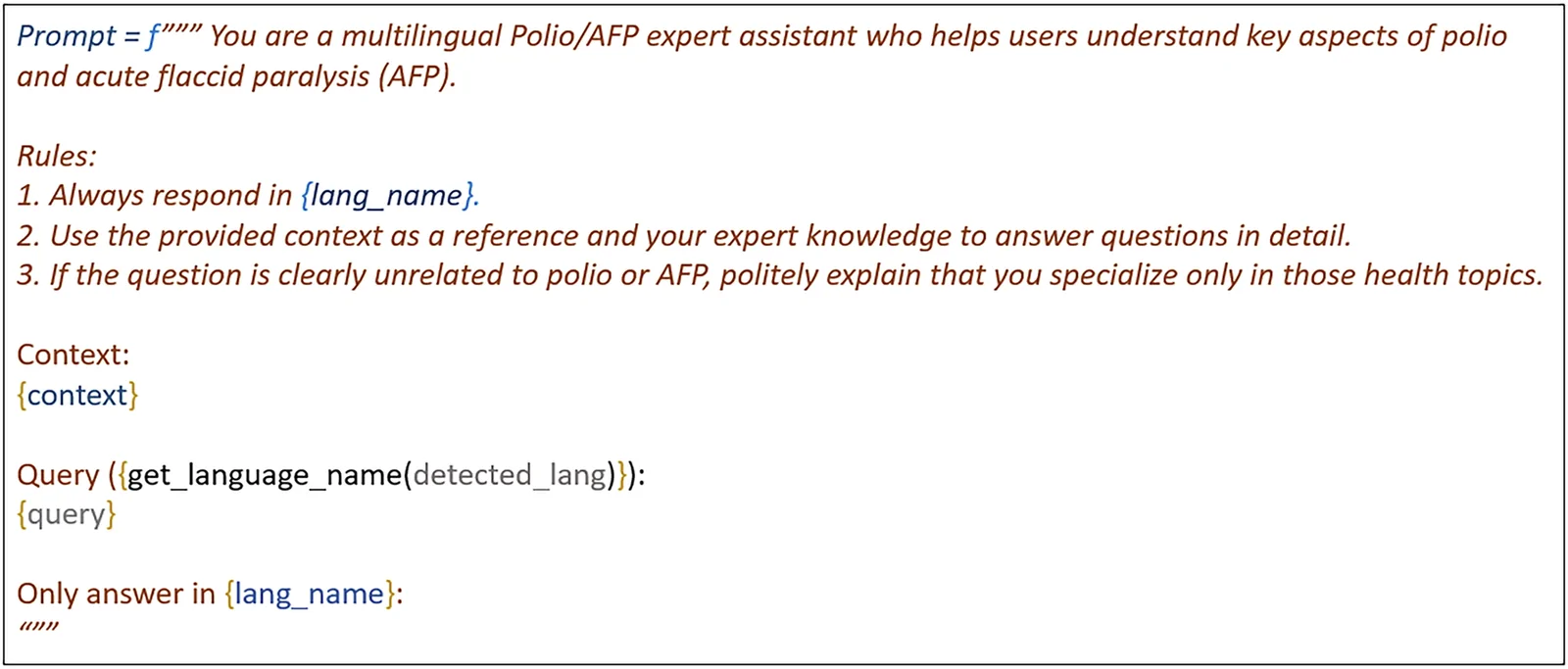
Structured system instruction and retrieval prompt designed for the AFP Assistant. It presents the structured system prompt designed to control chatbot behaviour during response generation. The instruction framework defines the chatbot’s role, multilingual behaviour, domain restrictions, and language detection capability.

As presented in Fig. 15, the system operates under a structured system instruction and retrieval prompt template specifically designed for this research. The system instruction defines the assistant’s role, multilingual response behavior, domain-specific scope, and language-handling capabilities. The retrieval prompt template further guides the response generation process by incorporating context retrieved from the knowledge base. By integrating these tailored instructions, the chatbot can consistently detect the user’s language, generate accurate and contextually relevant polio-related responses, and maintain reliable behavior within its defined domain of expertise.

### Multilingual support and detection of languages

**Fig. 16:**
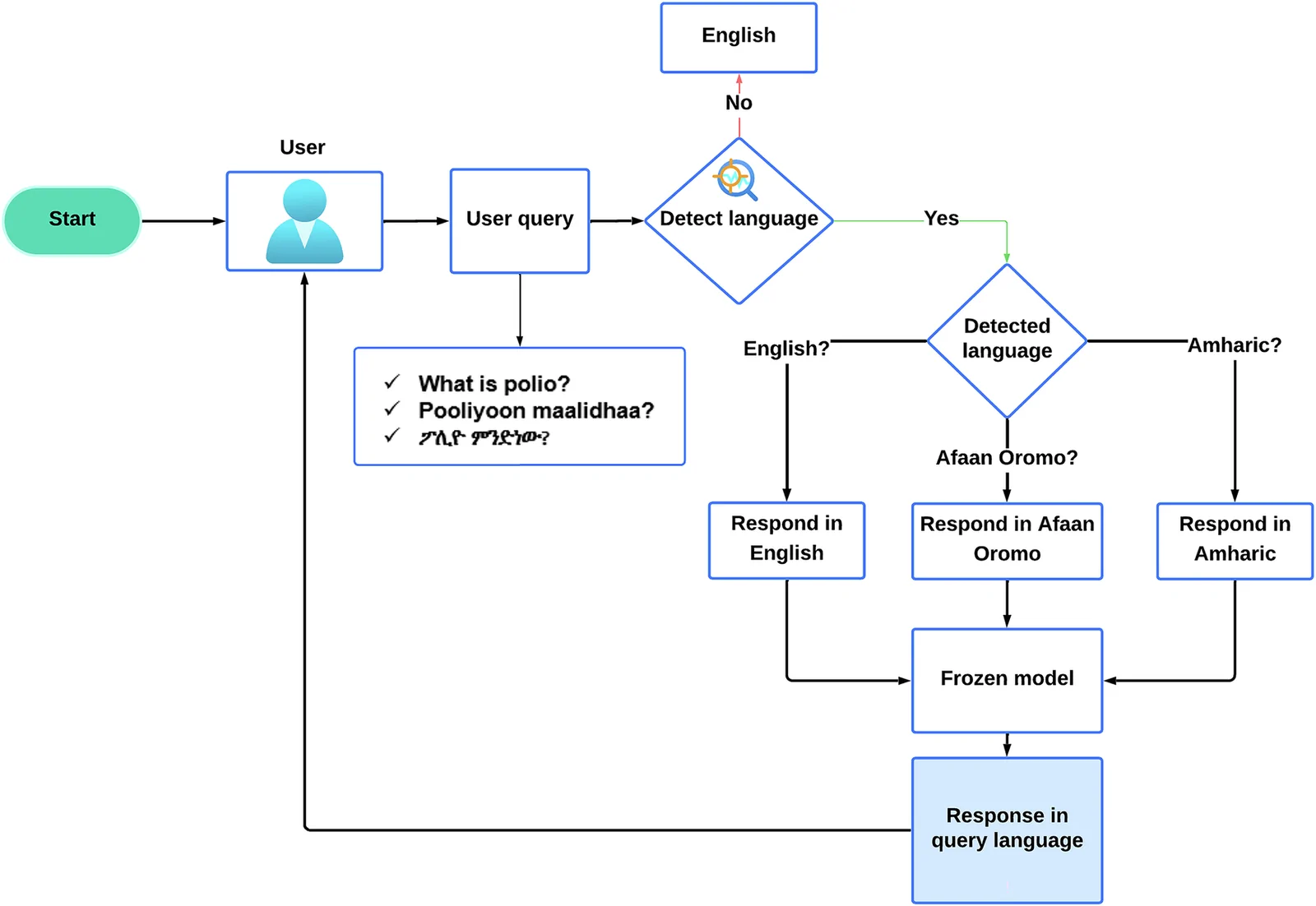
Flowchart showing the AFP Assistant query handling process. The user submits a query, which is processed by a language detection step. Based on the detected language, English, Afaan Oromo, or Amharic, the system responds in the corresponding language using a frozen model to generate the reply. Examples of user queries in different languages are included.

To ensure accessibility and maximize impact across Ethiopia’s linguistically diverse polio-affected communities, the AI system provides robust multilingual support in Amharic, Afaan Oromo, and English, eliminating the need for external translation layers that could otherwise increase response time. Query processing begins with automatic language detection, directing user inputs to the corresponding language-specific retrieval components within ChromaDB. A two-stage detection pipeline enhances accuracy and responsiveness: first, FastText^65^, trained on over 157 languages, performs initial detection after text preprocessing and demonstrates particularly strong performance for Amharic due to its subword-level modeling. When FastText’s confidence is low, the system invokes Compact Language Detector 2 (CLD2)^66^, which probabilistically reassesses the input and excels in handling shorter or noisier text, especially in Afaan Oromo. If neither detector confidently identifies the input language, the system defaults to English. By combining FastText’s speed and reliability for Amharic with CLD2’s precision for Afaan Oromo, the system efficiently handles multilingual and noisy queries while ensuring accurate, language-specific retrieval. The system further accounts for linguistic variability in real-world health communication. Amharic and Afaan Oromo exhibit rich morphological structures and flexible word formations, leading to diverse query expressions. FastText’s subword representations help capture these variations, while CLD2 enhances robustness to irregular and fragmented inputs. In addition, locally collected question–answer pairs enrich the training data with common community expressions and informal phrasing. Together, these design choices improve the system’s ability to interpret diverse inputs and generate contextually appropriate responses in multilingual community health settings. This workflow is illustrated in Fig. 16 below.

### System evaluation framework

The chatbot’s response accuracy was evaluated using a combination of in-house (developer) assessment and expert review. Generated responses were compared against verified information from the curated dataset used to construct the system’s knowledge base. In this study, response accuracy is defined as a multi-dimensional measure reflecting the extent to which a generated answer aligns with the factual content of the reference dataset while remaining contextually appropriate, clear, and informative. To operationalize this definition, a structured evaluation rubric was applied using a 5-point Likert scale (1 = very poor, 5 = excellent) across six criteria: factual correctness, completeness, clarity and understandability, level of detail, structure and organization, and contextual relevance (see Table 4). A predefined multilingual test set (Amharic, Afaan Oromo, and English) was used for evaluation. Six domain experts fluent in the target languages independently assessed the responses, and final scores were computed by averaging their ratings. This framework provides a transparent and reproducible approach for assessing response accuracy across multiple dimensions.

**Table 4:** Response accuracy evaluation rubric

Criteria	Definition	Level 1	Level 2	Level 3	Level 4	Level 5
Factual correctness	Alignment with verified AFP/polio medical information	Mostly incorrect or misleading	Major factual errors	Partially correct with inaccuracies	Mostly correct with minor issues	Fully accurate and consistent with reference data
Completeness	Coverage of all key aspects of the query	Very incomplete	Missing major components	Covers some important points	Nearly complete	Fully comprehensive
Clarity and understandability	Ease of understanding for general users	Very unclear or confusing	Difficult to understand	Moderately clear	Clear and understandable	Very clear, simple, and user-friendly
Level of detail	Adequacy of explanation without being vague or excessive	Too brief or too vague	Insufficient detail	Adequate but limited detail	Good level of detail	Well-detailed and informative
Structure and organization	Logical flow and formatting of the response	Poorly structured	Weak organization	Acceptable structure	Well-organized	Highly structured and easy to follow
Contextual relevance	Appropriateness to the user query and context	Irrelevant	Mostly irrelevant	Partially relevant	Mostly relevant	relevant and context-aware
Domain specificity	Alignment with AFP surveillance guidelines and public health practices	Not aligned	Weak alignment	Partial alignment	Mostly aligned	Fully aligned with domain standards
Local language response quality	Ability to understand and generate coherent responses in Amharic and Afaan Oromo	Not understandable	Poor fluency and meaning	Understandable with errors	Mostly fluent and clear	Fully fluent, natural, and accurate

To ensure an unbiased assessment, the evaluation was conducted on a strictly held-out test set that was not used during system development, tuning, or iterative refinement. The test set was constructed based on predefined criteria to ensure diversity and representativeness, including coverage of multiple languages (Amharic, Afaan Oromo, and English), variation in query complexity, and inclusion of common user intents observed in real-world deployment scenarios. This design allows the evaluation results to approximate the system’s true generalization performance and provides a more realistic estimate relative to tuning accuracy. Performance results were further stratified by language and by representative task categories, including AFP symptom-related queries and vaccination information. Although the system is not explicitly designed for task classification, these categories reflect practical usage patterns and enable more granular analysis of system performance across different types of queries.

To complement the expert-based assessment, a preliminary user-centered evaluation was designed as part of the broader Haqila platform deployment. The AFP Assistant is part of Haqila^67^, a unified AI-based platform designed to support CBS was implemented across eight sites in the Jimma Zone, Ethiopia, involving approximately 32 participants. Using purposive sampling, three sites, Seka, Jimma University General Hospital, and ShGGH, were selected based on proximity to facilitate engagement and feedback collection. Eight representative users were recruited to ensure diversity in gender, professional roles, and language proficiency. Participants interacted with the chatbot within routine workflows and provided structured feedback on usability, clarity, and contextual relevance.

### Ethical considerations and data security

Ethical approval for the data collection component of this study was obtained from the Jimma University Institutional Review Board (IRB) (Ref. No. JUIH/IRB/275/25; approved April 12, 2025). All participants involved in the interview process were informed about the purpose of the study, and informed consent was obtained before participation. The questionnaire was designed to assess community awareness and misconceptions regarding polio and did not collect any personally identifiable information or individual health records.

To ensure privacy and secure data management, the system utilizes Supabase as the backend infrastructure, which provides built-in support for authentication, secure data access, and user management. Supabase Auth is used to manage user authentication, with passwords securely hashed and never stored in plain text. Role-based access controls restrict access to user data and system resources to authorized components and administrators only. To further protect user privacy and ensure ethical data handling, the system records user queries in an anonymized manner, capturing only the query content, associated email, and submission date. No personally identifiable information is shared with third parties, and all recorded data is used exclusively for system improvement, aggregated analysis, and public health research purposes. These measures support the responsible handling of user interactions and promote the ethical deployment of AI-based health information systems in community settings.

### Experimental settings

This study involved five experiments designed to evaluate the performance of the AFP Assistant and its underlying architecture. The first experiment focused on model selection, where four candidate LLMs (Gemini 2.5 Flash, Gemma 3, LLaMA 3.1, and DeepSeek-R1) were benchmarked using a standardized set of five queries in English, Amharic, and Afaan Oromo. The evaluation examined each model’s ability to automatically detect the query language, generate accurate responses in the same language, and demonstrate semantic correctness in polio-related contexts. The second experiment addressed fine-tuning, where Gemini 2.5 Flash, selected as the base model, was trained on a curated dataset of 468 Q&A pairs (~130,248 tokens), consisting of 268 in English, 100 in Amharic, and 100 in Afaan Oromo. Fine-tuning was conducted on Google Vertex AI using configured hyperparameters (learning rate multiplier of 5, adapter size of 4, and 15 epochs), and performance was tracked through accuracy and loss metrics across training sets to ensure convergence and mitigate overfitting. The third experiment evaluated the RAG pipeline, with performance measured through visualization metrics, including relevance distribution scores, the average number of retrieved documents per query, and the mean query response time. Building on this, the fourth experiment tested RAG-grounded responses by verifying that all retrieved outputs originated exclusively from the curated knowledge base. This evaluation was conducted in Amharic, English, and Afaan Oromo, confirming correct indexing and consistent top-k retrieval with *k* = 7 in each language. The fifth experiment involved a comparative evaluation of SOTA models ChatGPT, Grok, and DeepSeek against the developed end-to-end functionality of the AFP Assistant through its dedicated user interface, assessing performance within a real-time interactive environment. Using identical polio-related queries, outputs were assessed qualitatively, with particular attention to accuracy, multilingual competence, and factual grounding. Accuracy was evaluated by comparing the system’s responses with verified reference answers, measuring the proportion of correctly identified and contextually relevant responses.

## Supplementary information

Supplementary information

## Data Availability

The datasets used in this research support both RAG and model fine-tuning. Both datasets are publicly accessible in the AFP_RAG repository on GitHub (available at [https://github.com/Gelane-biru/AFP_RAG]. For the RAG component, a dedicated document folder contains curated, translated, and validated multilingual question–answer pairs in Amharic, Afaan Oromo, and English, organized in separate files for each language to build a robust knowledge base. For model fine-tuning, a dataset folder hosts the validated multilingual dataset in JSONL format, structured for supervised training. All sensitive community-level information has been carefully anonymized to ensure participant confidentiality.

## Code availability

The underlying code used in this study, encompassing all components related to RAG and integration workflows, is publicly available in the same GitHub repository as the dataset (accessible at https://github.com/Gelane-biru/AFP_RAG) to ensure full transparency and reproducibility.
