# Supplementary material for: AFP assistant: a retrieval-augmented generation and large language model-powered multilingual polio chatbot for low-resource language communities
Source: NPJ Digit Med. 2026 Jun 30;9:804. doi: 10.1038/s41746-026-02944-4 (PMC13686964; doi:10.1038/s41746-026-02944-4)
Supplement: Supplementary file 1 [file 41746_2026_2944_MOESM1_ESM.pdf]

Supplementary material 1

Questions used for RAG evaluations across all languages: Amharic, Afaan Oromo and English respectively

Amharic

Q1. ፖሊዮ ምንድን ነው?

Q2. ፖሊዮ የሚከሰተው በምን ምክንያት ነው?

Q3. ፖሊዮ እንዴት ይተላለፋል?

Q4. ፖሊዮ ቫይረስ ወደ ሰውነት ከገባ በኋላ ምን ይከሰታል?

Q5. የፖሊዮ ምልክቶች ምንድን ናቸው?

Q6. የልጅነት ልምሻ ምንድን ነው?

Q7. የልጅነት ልምሻ ክትትል ፖሊዮን ለማጥፋት ለምን አስፈላጊ ነው?

Q8. የፖሊዮ ቫይረስ ዓይነቶች ምንድን ናቸው?

Q9. ከክትባት የተገኙ የፖሊዮ ቫይረሶች (VDPV) ዓይነቶች ምንድን ናቸው?

Q10. የፖሊዮ ኢንፌክሽን ደረጃዎች ምንድን ናቸው?

Afaan Oromo

Q1. Dhukkubni poliyoomayilaayitii (pooliyoo) maali?

Q2. Maaltu Pooliyoo kaasa (fida)?

Q3. Pooliyoon akkamitti daddarba?

Q4. Vaayirasiin pooliyoo erga altakkaa qaama seenee booda maal ta'a?

Q5. Mallattooleen pooliyoo maal fa'i?

Q6. Qaamni Akka Tasaa Laamsha’uun maali?

Q7. Hordoffiin AFP pooliyoo balleessuuf maaliif barbaachisa?

Q8. Gosoonni vaayirasii pooliyoo maal fa'i?

Q9. Garaagarummaan vaayirasii pooliyoo madaqoo fi vaayirasii pooliyoo talaallii irraa argamuu maalii?

Q10. Gulantaan dhukkubbii pooliyoo maal fa'i?

English

Q1. What are the symptoms of polio?

Q2. How can AFP be prevented?

Q3. What is the treatment for polio?

Q4. Why is polio vaccination important?

Q5. What causes acute flaccid paralysis?

Q6. What is the polio vaccination schedule?

Q7. How to report AFP cases?

Q8. What are the polio symptoms in children?

Q9. What are the AFP surveillance guidelines?

Q10. What are the global polio eradication efforts?

Supplementary material 2

Data collection interview questionnaires

1. What is your profession? (Doctor / Nurse / Health Officer / Volunteer / Other)

2. How many years have you worked in health or community service?

3. What region/district do you serve?

4. What are the most frequent questions people ask you about polio?

5. Which myths or misconceptions do you commonly hear about polio?

6. What information do people usually misunderstand or find confusing?

7. In your experience, what are the biggest knowledge gaps the community has about polio prevention or vaccination?

8. What age groups or community groups seem to have the least knowledge about polio?

9. In your opinion, what type of information or data should be included in the chatbot to make it more helpful for the community?

Supplementary material 3

Question used for model selection in Amharic, Afaan Oromo and English respectively.

Amharic

1. ፖሊዮ ምንድነው?
2. ፖሊዮ እንዴት ይተላለፋል?
3. የፖሊዮ ምልክቶች ምንድን ናቸው?
4. ፖሊዮን እንዴት መከላከል ይቻላል?
5. ፖሊዮ አሁንም በስፋት የሚገኝባቸው አገሮች የትኞቹ ናቸው?

Afaan Oromo

1. Pooliyoon maalidhaa?
2. Pooliyoon akkamitti daddarba?

3. Mallattooleen pooliyoo maal fa'i?
4. Dhukubba pooliyoo akkamitti ittisu danda’amaa?
5. Biyyoota amma iyyuu poliyoof saaxilamoon kam fa'i?

English

1. What is polio?
2. How is AFP detected?
3. What are symptoms of polio?
4. Why is vaccination important?
5. How polio is transmitted?
